# Taming the chimera of hybrid work: a work design perspective on supervisors’ working characteristics and leadership demands in hybrid work settings

**DOI:** 10.3389/fpsyg.2025.1650717

**Published:** 2025-11-12

**Authors:** Christiane R. Stempel, Jan Dettmers

**Affiliations:** Institut für Psychologie, FernUniversität Hagen, Hagen, Germany

**Keywords:** hybrid work, work characteristics, supervisors, leadership demands, qualitative research, work design, content analysis

## Abstract

**Introduction:**

Hybrid work models have become increasingly common, changing supervisors’ own work characteristics and their leadership demands. Based on work design research, we aim to assess hybrid work characteristics of supervisors and the job demands associated with leadership in hybrid settings.

**Methods:**

We conducted 33 expert interviews with direct supervisors who work in hybrid work models. We asked them about their work characteristics and leadership demands, using content analysis to identify key deductive and inductive themes.

**Results:**

Regarding supervisors’ work characteristics, findings show that specific work characteristics like information management or extended availability are perceived as typical of hybrid work. Furthermore, organizational factors and technical prerequisites play a major role in the supervisors’ working conditions and their leadership. In terms of leadership demands, it became evident that work design is especially important in hybrid settings due to reduced possibilities for direct interactions. Beyond, the quality of direct leadership interactions in hybrid environments is perceived demanding when decoding communication or perceiving problems among employees. With respect to role modeling as a leadership demand, three topics emerged when supervisors reflected on their role in hybrid work: Issues of trust and control, establishing quality social relationships, and questions around self and staff care.

**Discussion:**

Our study contributes to a comprehensive assessment of work characteristics and specific leadership demands in hybrid settings and provides new insights for the theoretical discussion on work design. In practice, the results highlight the need to analyze and adequately design hybrid work characteristics for supervisors to enable them to perform their leadership tasks.

## 1 Introduction

Since the coronavirus pandemic in the early 2020s, flexible working hours and remote work locations have become more common, contributing to the rise of hybrid work models (Flüter-Hoffmann and Stettes, 2022; Kniffin et al., 2021). Entgelmeier et al. (2023) characterize the alternation between onsite and remote work as hybrid work settings, regardless of their specific design features. This alteration can be viewed at an individual level or team level, with the flexibility of working hours often playing an additional role when several people are involved (Bellis et al., 2024). Variation in work locations and times is linked to sets of different work characteristics (e.g., autonomy, availability of information, social interactions and support), placing new demands on organizations and coordination (Fan and Moen, 2023; Rudolph et al., 2021; Rudolph et al., 2021; Xie et al., 2019). However, similar to the mythical creature Chimera, which is composed of different animal characteristics, hybrid collaboration emerges from a unique blend of working characteristics that are still not well understood. Supervisors are faced with the challenge of taming this workplace chimera to optimally organize hybrid work for themselves and their employees. To prevent overwhelming supervisors and to optimally support them in managing hybrid work, a closer examination of the work characteristics and leadership demands in this context is necessary.

In past research on work design, the significance, change, and impact of work characteristics have mostly been viewed through the lens of employees (Fan and Moen, 2023; Oldham and Hackman, 2010; Parker and Grote, 2022). The same applies to hybrid work characteristics (Xie et al., 2019). While supervisors are recognized as key agents in shaping, digital and hybrid work (Antoni and Syrek, 2017; Cortellazzo et al., 2019), the job demands and resources at their disposal have often been neglected. Most studies tend to focus on specific working characteristics linked to certain leadership behaviors (Stein et al., 2020; Stempel et al., 2023). However, precisely because hybrid work involves unique configurations of work characteristics that differ from or expand settings that are shaped by fixed working hours and locations (Fan and Moen, 2023; Parker and Grote, 2022; Xie et al., 2019), it is essential to draw a more comprehensive picture for supervisors. Furthermore, previous research indicates that supervisors may be exposed to different work characteristics than their employees (Zimber et al., 2015; Nielsen and Taris, 2019), but what this means for hybrid work settings remains unclear. Thus, the first aim of our study is to explore the job demands and resources of supervisors in hybrid settings.

A key job demand that distinguishes supervisors from their employees is the assumption of leadership responsibility (cf. Zimber et al., 2015). In research, leadership responsibility is often operationalized as a simple “yes” or “no,” without specifying the diverse demands placed on supervisors. Thus, a differentiated picture of the demands associated with the role of the leader is still lacking (cf. Arnold and Rigotti, 2023). This is not surprising, as previous research has focused primarily on the impact of leadership on employees, but neglected its role as a job demand for supervisors (Arnold, 2017; Bakker et al., 2023). In this respect, hybrid work presents a specific set of leadership demands, such as increased coordination and communication requirements (Krehl and Büttgen, 2022). Therefore, in the second research question, we aim to broaden the scope by assessing key leadership demands in the hybrid context, after focusing on the demands and resources of leaders themselves as a foundation in the first research question.

To explore these two foci—supervisors’ own work characteristics and leadership demands directed toward employees—in hybrid work situations, we use the framework of the job demands resources theory (JD-R, Bakker et al., 2023). Studying work characteristics of supervisors and leadership demands from a work design perspective is still a relatively new research interest with sparse empirical findings. Thus, we chose a qualitative research approach and conducted semi-structured interviews to obtain a comprehensive picture of supervisors’ work characteristics and their leadership demands in hybrid work environments.

We contribute to existing research by zooming in on the different job demands, and resources in hybrid settings, offering a more nuanced picture of the work characteristics the supervisors face. Here, the qualitative approach and the explicit focus on supervisors adds fresh insights into the theoretical discussion around job demands and resources (JD-R; Bakker et al., 2023). Moreover, we expand the understanding on the nature of job demands by including leadership as job demand, a perspective that has not been sufficiently considered in the model so far. In practice, our results provide clear references for approaching the design of hybrid work for supervisors and their respective teams. A discussion of the specific leadership demands might help organizations to reflect and refine their strategies for hybrid work practices to better support supervisors in these environments.

## 2 Theoretical background

### 2.1 Supervisors’ work characteristics in hybrid work

The uniqueness of hybrid work lies in the specific combination of onsite and remote settings (cf. Parker and Grote, 2022; Rigotti et al., 2024; Ong and Johnson, 2023). Xie et al. (2019) identified boundlessness, multitasking, demand for constant learning, and non-work-related interruptions as specific hybrid work characteristics but did not differentiate between supervisors and employees. However, research indicates that work characteristics of supervisors differ from those of employees, for instance in terms of access to resources, intensity or quantity of stressors (Herttalampi et al., 2022; Skakon et al., 2011; Zimber et al., 2015). However, while new constellations of different hybrid work characteristics are likely (Fan and Moen, 2023; Parker et al., 2017), a comprehensive assessment of supervisors’ work characteristics is still lacking.

To explore work characteristics of supervisors in hybrid work settings, we rely on the classification of job demands and resources as outlined by the JD-R model (Demerouti et al., 2001). Job demands are challenging and require continual effort, whereas the resources are empowering and motivating aspects of the work (Bakker and Demerouti, 2007). The main propositions of the model include a motivational and a strain path. The first explains how job resources are related to motivation and, consequently, to favorable outcomes, whereas the second describes how job demands that persist over a time can lead to strain and unfavorable outcomes (Bakker and Demerouti, 2007). In a comprehensive review, the authors of the JD-R discuss extensive empirical evidence and additions to the model (Bakker et al., 2023). As it provides a framework for a basic classification of work characteristics, the JD-R model seems suitable for exploring the job demands and resources in hybrid settings as a starting point for work design research in this context. In our study, we will therefore draw on JD-R to examine the following research question (RQ):

*RQ1*: What are the job demands and resources that characterize work for supervisors in hybrid work settings?

### 2.2 Leadership as job demand in hybrid work settings

Leadership demands are specific job demands that are associated with the responsibilities of the leader or supervisor role. Some are formalized in job descriptions, but others are more a set of varying expectations toward the supervisor that they are not always aware of (cf. Cloutier and Barling, 2023). The uniqueness of a leadership demand lies in its bidirectionality, as it is relevant both as a job demand for the supervisor and as an action directed toward the employees. Furthermore, as previous research has shown, performing leadership is not independent of the work characteristics of supervisors (Stempel et al., 2023). According to the conservation of resources theory (COR, Hobfoll et al., 2018), job resources can stimulate cycles of resource gain that provide supervisors with the necessary reserves to handle leadership responsibilities (cf. Stein et al., 2020; Stempel et al., 2023). For instance, a high degree of autonomy in hybrid settings might help a supervisor to engage in strategic planning for communication with her or his team. In contrast, a lack of resources or the threat of a resource loss can trigger resource loss cycles (Hobfoll et al., 2018). As Sherf et al. (2019) show, managers who were confronted with a high workload tended to treat their employees less fairly. The example by Sherf et al. (2019) indicates that the implementation of leadership demands requires an investment of resources or effort from the supervisor and can therefore be considered a demand in terms of the JD-R model (Dóci et al., 2020; Stempel et al., 2023).

Examining leadership demands from a work design perspective is a novel approach. It provides specific opportunities to shape leadership tasks in response to rapidly changing work characteristics (Herttalampi et al., 2022; Nayani et al., 2018). Hybrid work might evoke a demand for specific leadership. For example, increased flexibility is often linked to indirect forms of control that depend more heavily on management by objectives. These systems must be carefully designed to prevent negative consequences (Deci et al., 2016).

To comprehend and systematize the variety of leadership demands in a hybrid environment, it seems helpful to organize them along the three mechanisms through which supervisors exert influence. First, supervisors influence their employees by designing their work characteristics, such as offering autonomy, organizing work or distributing work tasks (Nielsen and Taris, 2019; Stempel et al., 2023; Vincent-Höper and Stein, 2019). Research indicates that the indirect influence via the design of work characteristics might have been underestimated in the past (Arnold, 2017; Schaufeli, 2015; Vincent-Höper and Stein, 2019; Yizhong et al., 2019). In comparison, there is extensive prior research on the second mechanism: the direct interactions between supervisors and their employees. This relational aspect predominately focuses on leadership behaviors like transformational or destructive leadership (e.g., Arnold, 2017; Mackey et al., 2021; Montano et al., 2023). And third, supervisors are role models for their employees, providing orientation and conveying the norms and expectations of an organization (Koch and Binnewies, 2015; Kranabetter and Niessen, 2017; Stempel and Siestrup, 2022). The latter presupposes that they reflect on their own leadership role, their expectations as well as the goals and norms of their team and organization (Haslam et al., 2011). While there is a wealth of research findings on all three mechanisms, the extent to which the specific leadership demands are shaped by a hybrid context remains largely unexplored. Given this gap, we structure our second research question along these mechanisms to explore whether specific leadership demands are linked to hybrid leadership:

*RQ2*: What leadership demands do supervisors associate with hybrid work?

## 3 Methods

### 3.1 Research design overview

We chose a qualitative approach and conducted semi-structured interviews to map the experiences of the supervisors in hybrid work settings, because comprehensive findings on work characteristics and leadership demands are still lacking. The interview guide contains general questions about hybrid work settings and the understanding of the own leadership role as well as a systematization of work characteristics based on the recommendations of the World Health Organization (2022). To analyze the data, we conducted a content analysis according to Kuckartz and Rädiker (2022).

### 3.2 Participant recruitment

Before the start of the project, we submitted our research project to the university’s ethics committee and received approval for the study. Participation was voluntary, and we informed the participants about the purpose of the study, the procedures, and the anonymization of the data obtained. Additionally, supervisors consented to the interviews being audio-recorded and received no incentives or compensation for their participation. Recognizing that flexible time and location models are not possible or common in every industry, we intentionally approached supervisors who lead hybrid teams. Nevertheless, the participants of our study stem from diverse industries. We aimed to acquire a comprehensive picture of work settings that allows a certain degree of generalization across industries. Additionally, we only included supervisors who had direct leadership responsibilities and, therefore, regularly interacted with their employees. We stopped the acquisition once we considered the variety of industries and positions sufficient (in terms of data saturation) to provide insights into diverse work characteristics of supervisors in hybrid work settings.

### 3.3 Sample

We conducted expert interviews with 17 female and 16 male supervisors in Germany resulting in an overall sample of 33 participants. The supervisors were between 24 and 63 years old with an average of 40.39 (*SD* = 8.84) years. They had various occupational backgrounds, such as engineering, health care, or environmental protection (see Appendix A for supplementary materials). On average, the supervisors reported an overall leadership experience of 8.15 (*SD* = 6.08) years, a tenure in their current position of 4.90 (*SD* = 4.68) years, and responsibility for on average 13 to 14 employees (*M* = 13.58, *SD* = 19.49).

### 3.4 Data collection

Our research team comprises the (first) author as a senior researcher in work and organizational psychology, three master’s students in psychology and one master’s student in sociology. Before beginning data collection, the research team familiarized themselves with the literature on hybrid work and the interview guidelines. The guidelines were tested in a trial interview and checked for comprehensibility and adequacy. The four master’s students collected data between May and August 2022 contacting supervisors for one-to-one meetings via private and university professional networks. The interviews were conducted either through video call or in-person, based on the interviewees’ preference. The average duration of the interviews was 43 min, and the video image was not captured. All interviews were audio recorded, anonymized, and content transcribed by the research team. The coding of the material was undertaken by all members of the research team and included the number of the interview transcript, the initials of the interviewer and the marked beginning of the sequence in the MAXQDA 2022 file (VERBI Software, 2021; e.g. A1_SK_5).

### 3.5 Interview guidelines

The structure of the interview guidelines (see Appendix B for supplementary materials) was based on general recommendations for work characteristics, including psychosocial risk assessment (Dettmers and Krause, 2020; World Health Organization, 2022). The first part of the guidelines ensured a standardized introduction of the topic, terminology, and the proceedings. Additionally, demographic (age, gender) and organizational background variables (industry, tenure of supervisor in current position, overall tenure leadership, number of employees supervised) were collected before the audio recording started. The following part addressed general questions about the perceptions of the hybrid work situation, which was often linked to the circumstances of the (post-)pandemic situation due to the timing of the interviews.

After exploring general views on the hybrid work situation, the supervisors were asked about their understanding of their own role and leadership demands as supervisors of hybrid working teams. The next section dealt with questions about conducive and hindering work characteristics for themselves and for their employees. We used established guidelines from the World Health Organization (2022) and Dettmers and Krause (2020) to structure the interview questions around work content, work organization, working hours, social relationships, and work environment.

Participants completed an interactive ranking task at the end of the questionnaire that was not relevant to this study. Finally, we asked the supervisors about open points, additions, and their current state of mind. We informed them about the further procedure of the research project and the overall length of the interview was noted.

### 3.6 Data – analytic strategies

**Figure 1:**
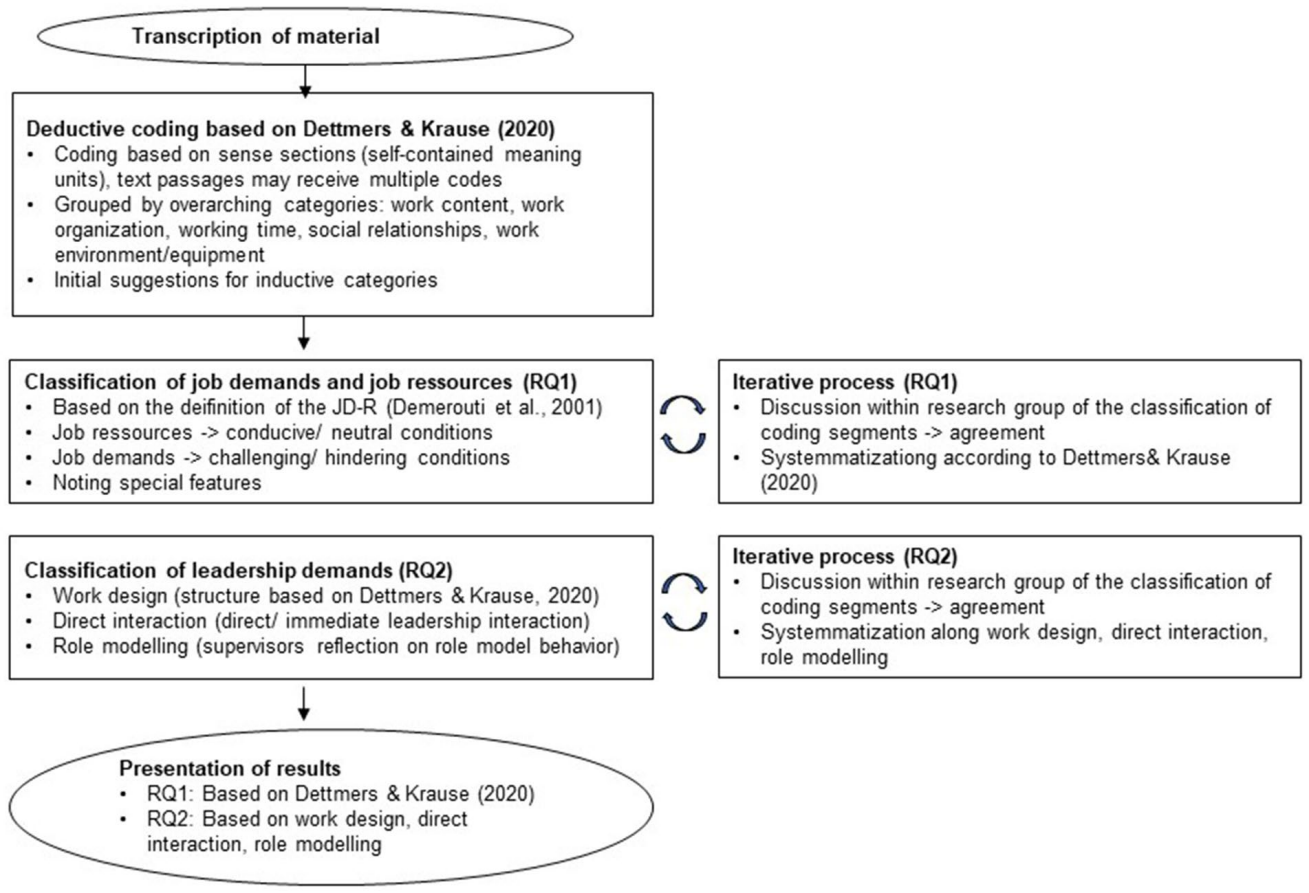
Process of coding procedure for research question 1 and 2 (RQ1, RO2).

The transcribed material was coded by the author and the research assistants (see Appendix C for the original quotations in German and their English translations). They coded the material based on sense sections (passages that form a self-contained meaning), whereby text passages could be assigned several codes. In the initial review of the study material, we coded work characteristics based on the deductive categories provided by Dettmers and Krause (2020) and Table 1 for conceptualization and anchor examples. Additionally, it was possible to derive and suggest initial inductive categories based on the material not covered by the classification of work characteristics by Dettmers and Krause (2020) and Figure 1.

**Table 1:** Codes of work characteristics based on Dettmers and Krause (2020) with anchor examples (references to hybrid work highlighted with*).

Work characteristic	Categorization	Code conceptualization	Anchor example from the interviews [code]
Work content			
Completeness of work task	Job resource	… means that the work includes tasks that demand preparation, organization, and evaluation in addition to the mere execution. Complete tasks are characterized by a mixture of automatized and complex demands.	Not coded
Autonomy*	Job resource	… comprises the degrees of freedom and of influence in terms of content and time in the work activity.	“Flexibility is good for me personally, of course. And of course, you can also change things a bit, change things in a different way in the team. When it comes to this switch to hybrid working, working from home, you simply have the opportunity to organize things differently.” [C4_LG_43]
Variability	Job resource	… means a variety of requirements and tasks.	“So, it’s definitely varied, simply because we do HR support from A to Z. There’s no boredom, because somehow nothing…, so of course there’s a core, but still a lot is different, so very diverse.” [C3_LG_39]
Emotional demands	Job demand	… includes emotional dissonance and emotional demanding situations such as dealing with grief or anger.	“However, what really weighs on me is my own responsibility toward my employees. Not that I cannot sleep at night, but I also take a lot of things home with me. I also wonder how is this person doing now, why did he or she react this way or that, did you perhaps express yourself incorrectly, did you step on his or her toes? For example, when things are said to me because I have built up a relationship of trust. And that bothers me. And that burdens me. But I think that’s also my job.” [B6_SDG_43]
Information overload*	Job demand	… means an excessive amount of information that exceeds the absorption and processing.	“I just think we have far too many inflow channels. You’re constantly being teased on teams. I find it really annoying. You get a lot of emails and then maybe even a WhatsApp or someone standing in the door and I have to say that I find this Teams chat in addition to Outlook unpleasant because you are always—and you know that psychologically- you are always being pulled out of your tasks. You always need time. So, it’s inefficient. It’s not productive.” [D6_EA_179]
Lack of information*	Job demand	… hinder employees in the execution of their tasks. Information may be missing, not up to date or presented unfavorably.	“Because it always goes hand in hand with the fact that you build up a certain information deficit. Especially if I now say that I work normally, I always alternate between 1 day at home and 1 day here, then this works. But if we are not on site for two or 3 days, I think it could become relevant, this information deficit that you gradually build up, if you do not get to hear about it on the floor, right?” [B2_SDG_55]
Qualification problems	Job demand	… occurs when activities do not match the existing qualifications or the person has not been trained adequately.	“I would say I really need a mentor. Someone who has really learned the ropes, who has professional experience in the field and who can teach you something. Most of it is just learning by doing and asking questions, reading through. There’s still a lot missing, which would be desirable to have someone on hand.” [C4_LG_67]
Underutilization of skills	Job demand	… refers to qualitative underload.	“There are a lot of things that do not really challenge me because the team is currently very understaffed. The marketing team actually consists of two people, myself and one full-time employee. The other colleague is a graphic designer, who then has other tasks. Then you have to do tasks that usually is done by the “ants.”” [B7_SDG_55]
Work organization			
Role ambiguity*	Job demand	… is given when responsibilities are unclear or non-transparent and when there are contradictory instructions.	“Well, it’s a balancing act, I have the greatest possible flexibility, so to speak, with vague specifications. Yes, what I just said. We basically have an obligation here at the plant that anyone who can work from home should do so and preferably as much as possible to limit the risks. At the same time, I should also be present, and I should be present less.” [B4_SDG_83]
Work interruptions/Multitasking*	Job demand	… refers to the disruption and unforeseen interruption of one’s own workflow as well as working on different tasks at the same time, between which one must switch back and forth.	“In this respect, Slack is a pretty good representation of office work. You’re constantly distracted with questions or something, at least for a short time, and then you have to come back in and then you are still thinking internally, yes, should I answer right now or later, and then you have it in your head.” [C8_LG_41]
Work intensity*	Job demand	… includes both a high amount of work required and high complexity of the work task in relation to the time available (e.g., time pressure)	“So, a very clear time problem is that I’m in a lot of meetings due to my many functions. I’m actually in meetings half the week, you could say, depending on the week. I take some to-dos with me from almost every meeting, which then pile up. When I think about the impact on my team, the fact that I’m in so many meetings means that I’m not available. This means that people are waiting for feedback from me and cannot continue working. Yes, that’s difficult, especially during induction, especially in training situations. If I’m in a meeting for four hours and everyone is waiting for feedback from me and then you come out and have 36 emails that need to be answered right away.” [C4_LG_75]
Lack of communication*	Job demand	… refers to a lack of communication and cooperation opportunities (e.g., in isolated individual workplaces)	“As a result, I lose contact with interdisciplinary departments. This means that I always have to deal with the same contacts and colleagues with whom I work on similar topics again and again. But I do not even notice the ones that are outside this circle and sphere of influence. So, there are employees in our company that I have not spoken to or seen for two or two and a half years.” [A5_SK_64]
Working hours			
Lack of time for recovery*	Job demand	… means a lack of undisturbed breaks, rest and recovery times.	“At home, I take little to no breaks. Here, when I’m at work, I meet up regularly, so no, we go out to eat every day. I do not have that at home, either I do not eat at all or you do not really have that kind of hygiene. Then you somehow type a quick email while eating or talking on the phone, so (.) I personally do not have a good grip on that, let me put it that way.” [B5_SDG_50]
Poor work hours design	Job demand	… refers to unfavorable designed working hours, shift work, or boundary management.	“Then there was usually the repair shift on Sundays and, in principle, I had my company cell phone, yes,. with a few exceptions, relatively extensively at hand, yes. So I was actually always available. Um, yes. Is part of it.” [B2_SDG_83]
Overtime*	Job demand	… is given when working hours are too long.	“My overtime has also skyrocketed. I had to take a step back from time to time and just say no, this week we are going to take a hard cut. Even though I had the feeling that I could easily continue working for another 4 h, I sometimes said no, after 8 h it’s actually over. But even then, I had the feeling that this was becoming increasingly blurred in the management circle, I would say.” [C7_LG_21]
Predictability*	Job resource	… refers to predictability and plannability of working hours as well as participation options concerning the duration, position, and flexibility of working hours.	“My day has become more structured because one does not longer see each other so often or simply does not have the classic meeting points in the office.” [C1_LG_27]
Social relationships			
Social support	Job resource	… refers to support received from colleagues and supervisors.	“My personal aspiration as a leader is to manage matters as I have to, but if there is a situation that I cannot resolve because information is missing or something is on a scale where I cannot or should not decide on my own, I always know where the door is and I always get advice and support.” [B8_SDG_65]
Social stress	Job demand	… refers to conflicts with or aggressions from colleagues or supervisors.	Not coded
Feedback and recognition	Job resource	… refers to feedback and/or recognition for one’s work from supervisors, colleagues, or clients.	“There is another team leader here with us. She has been a team leader for a very long time, is very experienced and I also have a collegial exchange with her that I send every few weeks and bring back a few points where she also gives feedback. How she assesses it, for example, is also helpful for me.” [A6_SK_37]
Work equipment and work environment			
Work equipment*	Job demand	… refers to unsuitable or unreliable equipment or tools needed to fulfil one’s work (e.g. software, protective equipment).	“It was a bit difficult for us to create the framework conditions in the first place. In other words, within the department, ensuring that colleagues were given the appropriate devices to be able to work remotely. The fact that it was a design department meant that …, yes, the requirement was a bit higher because not every laptop can be used to design.” [B2_SDG_7]
Work environment*	Job demand	… refers to unfavorable physical, chemical or biological and ergonomic factors.	“You do not even have a proper office chair, I do not have one at home. It’s just a normal chair, a chair like from the dining room, so of course you notice the back problems. So, it’s this ergonomic workplace, where the employer naturally places more value on having a proper desk, a proper chair, sufficiently large screens so that you can see it, and that’s not the case at home.” [B3_SDG_25]
Organizational factors [inductive category, not based on Dettmers and Krause, 2020]			
Organizational support*	Job resource	… refers to support structures on the organizational level.	“We have an accompanying management coaching program that enables us to do this, I think every 2 months or every 6 weeks. There is always a session where we work on different topics, and they are often free. It’s about communication or addressing difficult topics, potentially also hybrid topics.” [C8_LG_21]
Organizational hindrances*	Job demand	… refers to barriers and hindrances posed by the organization.	“It’s been a tough battle over the last 6 months. This has been discussed frequently in our management committee. Simply because we do not understand the point. The home office worked, why do I have to force everyone back into the office now? […] And that is, this doggedness, sometimes it is such a tough battle until all leaders across the board say, yes, why do not we do it, it works?” [A1_SK_99]
Organizational change	Job demand	…refers to change processes taking place within the organization.	“No, people do not follow. Yes, and they all had something to do before they were involved with transformation, yes, and I think many employees in the company are simply unsettled, because it’s been going on for 3 years now, that another topic keeps coming up, another program comes up and at some point, the time has probably just come where you have to ask yourself: “how much longer will this go on? When will normal working life start again?”” [D7_EA_294]

We used the superordinate category emotional demands to merge the subcategories of emotional dissonance and emotional/social demands. Moreover, for social relationships, we used the superordinate categories that can be differentiated into target groups such as supervisors, colleagues, or clients.

#### 3.6.1 Research question 1 (RQ1)

For this study, we followed an iterative coding process to address the two afore-mentioned research questions. Based on the JD-R framework (Demerouti et al., 2001), the work characteristics were abstracted to conducive (job resources) and challenging/hindering (job demands) characteristics. Following the JD-R model’s definition of job resources (Demerouti et al., 2001), we coded statements as “job resource” that described work characteristics as conducive or were neutral about work characteristics that are considered resources in the literature (e.g., variability; Dettmers and Krause, 2020). We coded statements as “job demands” when they were considered challenging or hindering as well as when they neutrally described work characteristics that are seen as stressors or demands in the literature (e.g., emotional dissonance).

It is important to note that the supervisors sometimes classify the lack of demands or stressors as a resource or vice versa. We incorporated this in our coding and highlight particularities in the discussion. Nevertheless, we reported the results according to the systematization of job resources and demands proposed by Dettmers and Krause (2020) and Table 1 for an overview of the concepts. The systematization is based on the overarching categories of work content, work organization, working time, social relationships, work equipment and work environment. Additionally, we built an inductive category to capture working conditions that relate to the organizational context (cf. World Health Organization, 2022). In an iterative coding procedure, job demands, and resources were discussed, summarized, and abstracted for a systematic analysis to identify particularities of hybrid work characteristics. The results of the content analysis are based on the frequency of codes across all interviewed supervisors.

#### 3.6.2 Research question 2 (RQ2)

To analyze the leadership demands that supervisors report with respect to hybrid work, we categorized the statements along three mechanisms by which supervisors influence their followers: first, by designing the work of their employees (Nielsen et al., 2008; Vincent-Höper and Stein, 2019); second, by direct leadership interactions (e.g., Arnold, 2017) and third, by being a role model to their employees (Koch and Binnewies, 2015; Kranabetter and Niessen, 2017). For the first category of work design, we applied the same structure that we established for the work characteristics of the supervisors themselves. Under the second category, we categorized all statements that included a direct or immediate leadership interaction. The third category included statements in which supervisors reflect on their role model behavior. Again, we chose the same iterative approach discussing the codes within the research team until reaching agreement on the coded segments. The results reflect the most frequently mentioned codes in the interviews.

## 4 Findings/results

### 4.1 Research question 1: Leaders’ job demands and resources in hybrid work settings

In the following, results of leaders’ job demands and resources in hybrid settings are reported along the deductive categorization system and the inductive category with anchor examples listed in Table 1 highlighting work characteristics linked to hybrid work with *.

#### 4.1.1 Work content

With respect to work content, participating supervisors reported autonomy as the most frequent resource and established a clear connection to the hybrid work setting. The supervisors appreciated the freedom to organize their work according to their own preferences (Table 1: C4_LG_43).

In terms of job demands, supervisors reported insufficient information management as common challenge in hybrid settings. Supervisors mentioned both, information overload (Table 1: D6_EA_179) but also a lack of information (Table 1: B2_SDG_55).

We also identified statements about work content that contradict the common conception as a job demand or resource. For instance, one supervisor states that hybrid work reduced emotional demands in online situations for him:

*“Inappropriate behavior is a completely different matter in real life than virtually. If I now think about what inappropriate behavior could be, hypothetically speaking, someone is somehow making a fool of you,* i.e.*, a candidate or customer is somehow making a fool of you, that’s not nearly as unpleasant in a virtual video call as it would be face-to-face. So, really face-to-face. Of course, that cushions it a bit.”* [C6_LG_59]

#### 4.1.2 Work organization

Supervisor statements on the job demands subsumed in work organization show a direct connection to hybrid working. For instance, supervisors described role clarity as a general resource but established a clear connection to the hybrid setting when talking about challenges due to role ambiguity (Table 1: B4_SDG_83).

Work intensity was the job demand that a large majority of the participating supervisors found challenging, and they often mentioned it in relation to either the hybrid work setting or perpetual change. For instance, one supervisor depicted an increase in meeting density causing time pressure (Table 1: C4_LG_75). In terms of work interruptions, supervisors described them as a major general job demand. On the one hand, supervisors made references to the hybrid setting, often describing interruptions due to attending multiple communication channels (Table 1: C8_LG_41). On the other hand, supervisors explained the possibility of working outside the workplace as a major advantage of hybrid work, because they are less interrupted and can use the time for focused work:

*“In the office, more often now [referring to interruptions], when there are more people there, you are also interrupted more often. Working from home is actually less in comparison. If you take yourself out of the phone line and if you have thought everything through, you are not interrupted as often. It’s actually better, yes.”* [B7_SDG_67]

Concerning communication and cooperation, supervisors, on the one hand, report less personal contact due to the reduced exchange opportunities in hybrid work settings (Table 1: A5_SK_64). On the other hand, some supervisors underline the better responsivity based on technologies applied in hybrid settings:

*“And thanks to these new communication options, such as Teams, the connection is always perfect to the company. No matter who has something, you can always get in touch, you can always provide support. It works really well.”* [A3_SK_5]

#### 4.1.3 Working hours

With respect to working hours in hybrid settings, supervisors perceived generally more job resources than demands. Time gains due to, e.g., reduced commuting (B2_SDG_55: “*So basically a time saving. The fact that you. that I save myself the drive*.”) and increased flexibility with respect to working hours and locations, were among the most frequently mentioned resources (B7_SGD_13: “*So, I’m totally flexible. I adapt to what’s going on at the time.”*). In this context, participants reported a better life domain balance in hybrid work settings, were they have the chance to work from home at times:

*“If you work remotely, I’d say you can make it a bit, yes, nicer so that you have a bit more time left for yourself. That’s definitely the biggest advantage for me.”* [B2_SDG_55]

Additionally, some supervisors experienced a better structure and increased plannability of tasks (Table 1: C1_LG_27). Job demands related to working hours, as for example overtime, are often mentioned in relation to work intensification (Table 1: C7_LG_21). Moreover, participants reported extended availability and insufficient recovery as a job demand, which was more pronounced due to the hybrid work situation (Table 1: B5_SDG_50).

#### 4.1.4 Social relationships

In terms of social relationships, participants did not establish a direct link to hybrid work but saw the points as general resources. Noticeable is that supervisors regarded lack of support (C2_LG_5: “*[…] when you get so little guidance from your supervisor”*), and appreciation (A6_SK_53*: “I think there is a lack of appreciation. […] Why have you forgotten us?”*) as hindering.

#### 4.1.5 Work equipment and work environment

Insufficient work equipment and work environment are seen as risks for occupational safety and health. In our study, supervisors described their equipment and their environment frequently as a resource for their hybrid work situation. For instance, in terms of equipment, one supervisor praised the possibilities related to the usage of advanced technology:

*“Sharepoint and OneDrive allow documents to be shared or screens to be shared more easily than was the case in the past. Instead, you used to send documents back and forth to each other and say, on page so and so section so and so, which was much more complicated.”* [A5_SK_54]

Nevertheless, in other cases inadequate equipment (Table 1: B2_SDG_7) and work environment (Table 1: B3_SGD_25) was considered a hindering demand.

#### 4.1.6 Organizational factors (inductive category)

Organizational context factors are not included in the systematization offered by Dettmers and Krause (2020). Supervisors named a range of organizational job resources in hybrid work settings. For instance, two supervisors mentioned a small team size as advantageous for working hybrid. Moreover, participants reported support structures ranging from the provision of helpful guidelines and consulting services to training and learning opportunities (Table 1: C8_LG_21). Participants considered the lack of support in the above areas, for instance in training, as a significant job demand (A5_SK_54: “*No, it’s just that there was no training in advance on how to lead an employee at a distance.”*).

Additionally, some supervisors reported that aspects of the organizational culture were hindering factors in their hybrid work setting (Table 1: A1_SK_99). Demands associated with organizational change were not exclusively associated with hybrid work. Participants described permanent change processes that sometimes overlapped and were seen as additional demands for themselves and their employees (Table 1: D7_EA_294).

In summary, not all work characteristics are equally relevant for hybrid work (see Table 1). In terms of work content, autonomy and information management are most often related to hybrid work as well as the concepts regarding work organization. With respect to working hours, all aspects have been associated with hybrid work, with the exception of working hours design, which appear to be more general in nature. Social work characteristics of supervisors seem less specific to hybrid work. In contrast, work environment and work equipment as well as organizational support and organizational culture show specific characteristics in the context of hybrid work.

### 4.2 Research question 2: Leadership as a job demand in hybrid work settings

Below, the results for research question 2 are reported along the three mechanisms of leadership: work design, direct interaction. and role modeling. Anchor examples are listed in Table 2.

**Table 2:** Codes of leadership demands in hybrid work settings with anchor examples.

(Sub-)codes	Anchor example from the interviews [code]
Designing work for emloyees
Work content
Providing autonomy	“Virtual working naturally offers employees more autonomy, and I see that as a clear advantage. Once you have been trained, you can work relatively independently.” [C4_LG_41]
Information management	“Sometimes there are too many channels for me because we cannot focus. This is also a recurring theme in the team routines, in the big team routines, where we say, friends, let us just make an information channel and then you read it when you have time to read something.” [C2_LG_39]
Handling emotional demands	“And you probably have to make sure that it does not become too much. With regard to my employees, especially one employee who has a. I think it’s very difficult in terms of the workload because the child is at home and she tries to do mobile work on the side. or does mobile work, well not now, she does not try. (embarrassed laughter) she does. she does it.” [B1_SDG23]
Work organization
Coordinating	“I have to deal with, um, the additional workload that I distribute and then I think in the end it’s about how intensively I get involved in the individual measures, how I hand over such topics. How intensively do I support such topics? And that is of course something that is much easier for me in person than it is now in the digital space. So, for me personally, it’s easier to sit down with someone somewhere for an hour or look at something on site than to write them an outlet.” [B2_SDG_93]
Designing communication	“Okay, yes, basically I first had to understand that it was more important to moderate communication a little. These conversations that we used to have in the office no longer took place and it was always a conscious call about conscious problems or questions and requirements that were there at the time, but all these nuances and intermediate levels were no longer there. You hung up and were done with the topic. We had to somehow reintroduce that and bring it about and it helped us that we simply turn on teams in between, let it run in the background, so to speak, everyone works, teams is still on, you get a bit of an idea of what the others are doing and a weekly jour fix, where we all sit together again and review the whole week and yes, we have now managed to do that well thanks to these mechanisms that we have developed.” [B8_SDG_7]
Providing role clarity	“It is important that the employees know what their tasks are, what the activities are, otherwise it will not work. Or not really well, because you simply have to be on site for certain things in order to control them. So, from that point of view, the individual’s job must be known and must be carried out safely, that’s the alpha and omega.” [B5_SDG_5]
Working hours	
Designing working time	“I often see a colleague, she works full-time. She’s often in the office because she says she cannot work well at home. […] And the other colleague, who is also part-time, is always there one day a week, so we are actually all there that day. We try to set that up, so on Tuesday, we always go through everything together.” [B7_SDG_17]
Boundary management	“I have noticed that I and my employees often work longer hours. So, you do not tend to draw the line like that. I can only encourage them to say, that’s okay, please organize your work so that you can manage it in the time available and otherwise it will just have to wait, just give me feedback.” [C8_LG_17]
Controlling working hours	“I try to be very, very strict with the trainee. That means that she takes breaks, that she takes regular breaks, that she does not work overtime. If she does work overtime, I make sure that she works it off within two weeks, that we are always on the same level. In other words, I check the recorded working hours to keep an eye on things. I also regularly check the other employees’ working time records and overtime accounts to keep an eye on what’s going on in terms of capacity and whether any relief is needed. We still have regular meetings where we talk about these things so that we know that nobody here is completely burnt out.” [C4_LG_29]
Social relationships	
Establishing social relationships	“Because the onboarding of trainees and dual students in new departments is currently much more difficult than in person. In some cases, young people are perhaps not so confident to call, write something or ask if something is unclear in the departments. In this respect, everyone would actually like to see more on-site work again and for the specialized departments to be more on-site, so that we can work together more closely and motivation is sometimes difficult anyway.” [D1_EA_25]
Facilitating social contact	“There are touchpoints for this topic of coordination, which I mentioned earlier, not just coordination on the content of tasks, but also coordination on questions such as “how am I doing,” do I have any issues that I would like to raise? So creating touchpoints is certainly a leadership challenge.” [C1_LG_29]
Problem solving	“We are now in a hybrid situation, which means that employees are back on site here 3 days a week and that we have to postpone conflicts or resolution discussions until we are on site.” [B9_SDG_3]
Designing feedback	“Getting feedback, giving feedback, giving each other praise because we work together very intensively in a wide variety of constellations, for example through a monthly team survey, but also through employee appraisals, feedback meetings, which have of course become much more important, especially in onboarding, and are also held in a more standardized and structured way.” [C6_LG_33]
Staff-care	“In other words, in the week in which she does not have this, this obligation for [note: picking up her child…] we said: “oh come on, a bit more 1 week and I’ll give you the other week home office in return.” So, this person would otherwise only have the chance to look for a job closer to work that allows these conditions. And since I do not want to lose this person, the company has accommodated her a lot. So, all in all, this means that I have a chance to respond to individual fates.” [A1_SK_99]
Work environment/equipment	
Facilitating an adequate work environment/equipment	“Desk chairs or something like that, so anyone can take them home at any time, or even the height-adjustable desk, if it fits in at home, so anyone who wants that, I’ve also approved it and we have also had three or four employees who have then taken their office chair home with them.” [D3_EA_29]
Direct leadership interactions	
Communication quality	“Well, in a face-to-face conversation you can react very differently and convey feelings very differently and so on. And if you want to type it out in words or whatever, it often leads to misunderstandings or it takes a lot of formulation work to really get things across the way you want to say them so that there are no misunderstandings.” [A5_SK_9]
Perception of problems	“[…] are they exhausted? Are they tired? Are they stressed? Are they nervous? Yes, if you see someone in the corridor or in the office next door, you notice that. If someone just is not there and you do not see them, then you do not necessarily notice it, because then people might also pull themselves together during a phone call and not be so open about it” [D6_EA_25]
Immediate feedback	“So, of course I can (um). What we just had as a topic, the communication, I can of course personally contribute to that. I can say, “Hey, great job! Great, great!.” I can do that in writing, I can call. I can do all that.” [B1_SDG_49]
Responsiveness	“In terms of leadership with regard to the team, you are also there faster, so you can be contacted faster, I say. Because it’s often just a click and you can get someone immediately, you have someone on the line immediately or you can chat with the team.” [B1_SDG_49]
Role modeling	
Reflecting one’s role	“And as a manager, I also find it difficult not to take on this controlling role. Because I do not think that’s appropriate, and I do not do that here either. But I still somehow try to keep everything together and make sure the work gets done.” [B1_SDG_15]
Recognizing own areas of responsibility	“It’s incredibly important to me that no one in my team goes home with burnout, because I’m the first one to knock on [supervisor’s name]'s door and say we cannot carry on with the same workload as before with twelve people.” [C2_LG_19]
Role modeling	“But everything that can be classically assigned, I usually record and that is also important to me, on the one hand to remain resilient in the long term and also because I find it difficult to set an example if you always show that you can only do it if you work overtime.” [C1_LG_39]

#### 4.2.1 Designing work for the employees

##### 4.2.1.1 Work content

Hybrid leadership demands regarding work content centered around the design of information. On the one hand, some supervisors highlighted that advanced technology makes it easier to structure information for their employees:

*“We have learned a lot during this time,* i.e.*, during the whole phase, to work with it and to do it digitally and to structure ourselves there and that always gives me as a leader…, I know, I can see how far something is, we have already become much more digital. That’s really. Yes, that’s a big advantage. It’s also much more useful in leadership if you know where things are and you do not have to call and ask and so on.”* [B7_SDG_37]

On the other hand, new challenges in information management arise such as avoiding information overload within the team (Table 2: C2_LG_39). Some participants in our study emphasized the importance of providing autonomy to their employees in hybrid work environments (Table 2: C4_LG_41). Two supervisors found it difficult to address emotional burdens that are associated with their employees’ preference to work from home (Table 2: B1_SDG_23).

##### 4.2.1.2 Work organization

With respect to work organization, all supervisors made statements that stressed coordination or communication tasks as essential in hybrid work settings. These two categories were the most frequently coded among leadership demands, including the following good practice example about improved coordination and communication:

*“Yes, the way I deal with my employees is a bit more organized. So, there are simply very specific weekly planning meetings, there is a tool for this, a tool that makes it easier […] … simply communicate and collaborate with each other at a distance.”* [C8_LG_27]

But even more often associated challenges such as the distribution of workload when not working onsite (Table 2: B2_SDG_93). Another example for designing communication was provided by a supervisor who explained how s/he re-structured communication in response to the hybrid setting (Table 2: B8_SDG_7). In addition, participants emphasized the clarification of roles as an important leadership task in general and particularly in hybrid work settings (Table 2: B5_SDG_5).

##### 4.2.1.3 Working hours

Participants described the importance of designing working hours in hybrid settings to enable face-to face meetings (B7_SDG_25: “*I make sure that when we are all in the jourfix… or have the jourfix, that we are all in the office.”*) or to optimize flexibility (Table 2: B7_SDG_17). In their statements, the supervisors noted that hybrid work was associated with the danger of blurred boundaries between their employees’ private and work life. Therefore, the need to design availability and enable detachment from work was perceived as even more relevant in hybrid working. Being aware of this, some supervisors still seemed to struggle with addressing this issue (Table 2: C8_LG_17).

Reduced visibility due to hybrid work was relevant for monitoring excessive working hours and whether employees were taking breaks. One supervisor described how closely s/he tried to control working time, especially for trainees (Table 2: C4_LG_29).

##### 4.2.1.4 Social relationships

The need to organize social relationships in hybrid work settings was often mentioned by the leaders. Reduced visibility and addressability of employees was considered challenging for exercising leadership aimed at fostering team spirit and social cooperation. For instance, one supervisor complained that he perceived less empathy among the team members in hybrid settings (B6_SDG_25: *“But empathy,* i.e.*, interpersonal things, are also very much lost in my view.”*) and another stressed the importance of creating touchpoints to stay in contact (Table 2: C1_LG_29). Therefore, supervisors thought about how to design feedback and social support in hybrid work settings (Table 2: C6_LG_33). In this context, onboarding and establishing social relationships was mentioned as a specific leadership demand that had become more challenging (Table 2: D1_EA_25). Further, the leadership demand of problem solving was perceived as a new quality, and some supervisors pointed out that they explicitly address conflicts and other difficult topics in person (Table 2: B9_SDG_3). At the same time, the participants in our study perceived it as a hybrid leadership advantage to be able to address the individual needs of their employees more easily due to the increased flexibility. For instance, one supervisor referred to a case in which s/he was able to successfully respond to an employee’s care-giving need (Table 2: A1_SK_93).

##### 4.2.1.5 Organizational context

With respect to organizing work equipment and the work environment for their employees, some supervisors mentioned their dependency on organizational policies or context factors. A positive example was given by one supervisor describing a general organizational arrangement regarding access to office furniture (Table 2: D3_EA_29), whereas another referred to problems with equipment provided for remote working (B4_SDG_83: *“I could lead better from a distance,* i.e.*, from a distance, if I, if the employees had better hardware. But that’s not possible because it’s not available.”*).

#### 4.2.2 Direct interactions

Concerning direct leadership interactions with employees, supervisors stressed difficulties arising from reduced face-to-face interactions. For instance, when interacting via intermediaries such as video or telephone calls, some supervisors described a loss of important information about the “soft factors” of communication (Table 2: A5_SK_9). Based on this reduced “quality” when communicating via an intermediary technical tool, participants described it more challenging to engage in staff care and provide support (Table 2: D6_EA_25). In terms of immediate feedback, some supervisors reported challenges (B2_SDG_61: *“With digital communication, it’s a different story. An email is not written as quickly as you just stick your head in somewhere or you meet at the coffee machine to get coffee.”*), while others believed that technical connectivity makes it easier (Table 2: B1_SDG_49). Additionally, some supervisors were particularly pleased with their increased responsiveness for the employees (Table 2: D3_EA_57).

#### 4.2.3 Supervisor role (modeling)

The participating supervisors reflected on several topics regarding their role as a supervisor in a hybrid work setting. However, three areas of insecurity were noticeable among the leaders. The first topic dealt with the relationships at work illustrating the wish to establish social resonance with and among employees while avoiding estrangement. Participants expressed concerns about establishing quality social relationships when communication was partly mediated digitally, and people saw each other less in person. One example vividly illustrates the wish for social resonance, reflected by the sensory oriented description of the communication, while expressing the fear of estrangement at the same time:

*“So, I perceive it as challenging because certain things cannot be replaced by technology. Let me take the example of emotions, sensing, feeling, the facial expressions and gestures of the other person, which are much more difficult to perceive via a screen. I also found it uncomfortable at first, because I have to say that my claim as a leader is precisely that I have these antennae. It was something we all had to learn to deal with. There was no other way. We could not leave people on their own.”* [B6_SDG_5]

Related to sustainable social relationships, the second topic centers around trust versus control. Reduced visibility demanded more trust on the part of the leader, while at the same time results had to be tracked and fairness among employees ensured. One supervisor described her struggle to find a balance between the two poles when exercising leadership (Table 2: B1_SDG_15). The issue of trust versus control alone indicates insecurities about the responsibilities of the own role as a leader. However, while problems regarding trust versus control affect the task-related level, questions regarding care address the person level. On the one hand, participants acknowledged staff care as an essential part of the leadership role (Table 2: C2_LG_19). On the other hand, supervisors struggled with the extent of their responsibility with regard to drawing boundaries. For instance, one statement showed that the supervisor understood employees possibly neglected breaks or exhibited excessive work behaviors that could be described as deliberate self-endangerment yet was helpless meeting staff care responsibilities:

*“Nobody is instructed to work outside normal working hours, not at all. I do not expect that here, and I do not expect that at home either. But if you want to do it because it’s beneficial for you, then you should do it. But of course, you then have less influence on whether the rest periods etc. are adhered to. You can of course point this out the next day if “You did not pay any attention to the rest periods” or “You worked more than 10 h in a row, that’s not possible!” but there’s not much more you can do.”* [B1_SDG_51]

In addition to reflecting on their own leadership role and responsibilities, we asked explicitly about the participants’ role modeling behaviors. Some supervisors had not thought about it (B6_SDG_13: *“In fact, I have not given much thought to how I feel in this situation.”*) or considered it not important when not working on site (B2_SDG_31: *“So, from my perspective, the health-oriented behavior in mobile working does not play or did not play a role for us.”*). Other supervisors gave examples showing that they were aware of their role modeling function in hybrid settings usually connecting it to topics like availability requirements (A4_SK_50: *“I do not send any emails at the weekend that I can still send out on Sunday evening at 9 p.m., because nobody reads them then anyway.”*), overtime (Table 2: C1_LG_39), or health behaviors (B10_SDG_24: “*I still make sure that I’m doing well and that I’m conserving my resources in this area. I’ve had problems with that in the past, so now I pay particular attention to making sure it fits.”*). Interestingly, several supervisors reported differing behavioral standards for themselves versus their employees. For instance, a contradictory statement revealed that the supervisor deliberately endangered himself while attempting to assert care responsibilities for his employees:

*“With the workload I have no limits for myself. I take a completely different approach with my colleagues. They finish at 18:00. It’s rare that I ask someone to help me or deliver something at 6 p.m. or after 6 p.m. Yes, I’m 100 % behind that. If people are ill, they should stay at home longer. So, I’m 100 % behind people recovering and having a good work-life balance. I could not care less about that. I’m a massive workaholic.”* [D9_EA_33]

## 5 Discussion

The aim of our study was to explore leaders’ work characteristics and the leadership demands they face in hybrid work settings. If we first look at the supervisors’ own work characteristics, it can be said that most work characteristics used to describe the general workplace also play a role in hybrid settings. In this respect, the chimera of the hybrid work seems to be composed primarily of creatures familiar to us. Nevertheless, our results show that certain aspects might be more pronounced in hybrid settings, suggesting a different weighting of job resources and job demands (see also Xie et al., 2019). The flexibility associated with hybrid work (Entgelmeier et al., 2023; Rudolph et al., 2021) is reflected in our findings regarding the perception of increased autonomy and predictability, but also work time demands like lack of recovery or overtime.

This tension between autonomy as a job resource and as a potential (temporal) job demand, which also entails health risks, has been discussed in previous research (Mazmanian et al., 2013; Väänänen et al., 2020). However, studies indicate that the negative consequences of autonomy cannot be attributed to autonomy alone, but are primarily due to the simultaneous increase in other job demands and thus depend on additional contextual factors (Kubicek et al., 2017; Dettmers and Bredehöft, 2020). These findings underscore the necessity of closely examining the constellations of job demands and job resources and their effects in hybrid contexts.

It is noteworthy that supervisors emphasize conditions related to work organization (role ambiguity, work intensity, work interruptions, and lack of communication) in the context of hybrid work, understanding them not only as a demand but also as a resource. In particular, the option to work remotely with greater focus and fewer interruptions, as well as increased responsivity, are often considered positive features of hybrid work (cf. Bellis et al., 2024). Not surprisingly, the assessment of a range of hybrid work characteristics, such as interruptions, lack of communication, information management, and work equipment, is significantly shaped by the access to, and quality of information and communication technologies (ICTs; cf. Bellis et al., 2022; Wang et al., 2020). Moreover, since supervisors operate at the interface between the organization and the employees, organizational support as well as hindrances determined their possibilities to design hybrid work. For instance, the provision of good hybrid work equipment and an adequate working environment for supervisors depends strongly on organizational preconditions. Thus, hybrid settings are characterized by a specific pattern of job demands and resources whose interplay and impact calls for further investigation (Ong and Johnson, 2023; Parker and Grote, 2022).

Based on the exploration of the work characteristics of supervisors, our second research question was dedicated to leadership responsibilities as a specific job demand for supervisors in hybrid settings. Our findings show a very nuanced picture of leadership demands in hybrid settings organized by work design, direct leadership interactions and role modeling (see Table 2). In general, an increase in flexibility concerning working time and location and reduced opportunity for direct interaction seem to necessitate targeted work design leadership (cf. Bellis et al., 2024). Studies on the task and people-oriented remote leadership in crises show similar results, stressing the need for coordination, communication, and the facilitation of social interaction (Bellis et al., 2022; Krehl and Büttgen, 2022). Zooming in on the variety of leadership tasks associated with work design shows that it is characterized by the specifics of the hybrid work arrangement that Xie et al. (2019) describe as reflecting the “increasingly diffusive, polychronic, disruptive and evolving nature of work” (p. 502).

Besides the leadership demands associated with work design, supervisors also perceive a different quality of direct leadership interactions in hybrid work settings. Reduced direct contact and visibility changes the way supervisors communicate and establish relationships (Bellis et al., 2022, 2024; Nayani et al., 2018). Our findings show that supervisors in hybrid settings seemingly need to invest more energy in decoding communication and perceiving problems among their employees. However, the tasks of providing immediate feedback and being responsive were sometimes considered simpler and sometimes more difficult in hybrid settings. One explanation for this could be the underlying contextual factors or the aforementioned need to plan and design social interactions in such a way that they meet the requirements of hybrid work situations. Another explanation could be the individual preferences and appraisals of the leadership demands by supervisors (cf. Ma et al., 2021; Mitchell et al., 2019).

In terms of reflecting one’s own leadership role and role modeling behaviors, three topics related to hybrid work emerged. First, supervisors expressed insecurity about the dimension of trust versus control. On the one hand, supervisors acknowledged that more trust was needed in hybrid settings because micro-managing was neither always possible nor useful. Especially, because employees cherished the autonomy that came along with flexible work characteristics (Stempel and Siestrup, 2022). On the other hand, there was responsibility for outcomes and performance. Here, some supervisors struggled to position themselves adequately. A second topic that emerged was the challenge to establish social resonance in hybrid settings and avoid social estrangement (see Rosa, 2016). Less overlap of working hours and locations among the team members made it increasingly difficult to establish sustainable social relationships and a team spirit or avoid feelings of loneliness (Bellis et al., 2022, 2024; Parker and Grote, 2022). The third dimension, which was the subject of the hybrid role reflection, was care versus self-endangerment. Self-endangerment as a set of excessive work behaviors has been associated with flexible work characteristics and adverse health effects (Deci et al., 2016; Yokoyama et al., 2022). In our interviews, supervisors were concerned about staff- and self-care in increasingly boundaryless work environments (Bellis et al., 2022; Xie et al., 2019). Reduced visibility and information quality in hybrid settings as well as an increased need for indirect control demanded extra effort to execute staff care and to avoid self-endangerment. In this context, reflections regarding the self-care of the supervisor or own excessive work behaviors played a role. For organizations, these topics seem to be relevant to address to (re)-define leadership roles and reduce ambiguity related to leadership responsibilities.

### 5.1 Implications for theory

Our findings provide new impulses for the theoretical discussion on the work characteristics of supervisors. First, we notice difficulties taking a look at the clear classification of job resources and job demands as proposed by JD-R (Bakker et al., 2023), because the individual appraisal seems to be decisive (cf. Ma et al., 2021; Mitchell et al., 2019). In our interviews, supervisors appraise a lack of job demands sometimes as a job resource or vice versa. For instance, supervisors in our interviews appreciated a well-functioning information flow as a job resource, particularly for their work in hybrid settings, whereas others described a lack of information or information overload as a classical job demand. This contrasts with the statement by Bakker et al. (2023) that “a low score on a job demand is not a resource” (p. 42) even though the authors acknowledge that different occupations have different job demands and resources. The challenge-hindrance framework addresses this problem of classifying job demands by offering a differentiation based on the valence of the job demands (Cavanaugh et al., 2000; Sonnentag et al., 2023). Nevertheless, in our study the appraisal not only concerns the nature of job demands but also job resources or a lack thereof. This finding aligns with a study on work characteristics and health, which demonstrated that low demands can reduce the health risk while low resources increase it (Dettmers and Stempel, 2021). Overall, we assume that not only occupations differ in their job demands and resources (Bakker et al., 2023), but also that work arrangements such as hybrid work or the supervisor position are composed of a specific pattern of high or low demands and resources (e.g., Parker and Grote, 2022; Rigotti et al., 2024; Xie et al., 2019). Thus, an analysis of working condition profiles in different work settings or roles might be a sensible approach to estimate consequences and initiate targeted work design (cf. Ong and Johnson, 2023). This is particularly relevant, because supervisors’ capacity to perform leadership is closely linked to their job demands and resources (Sherf et al., 2019; Stempel et al., 2023).

By investigating our second research question on leadership as a demand for supervisors in hybrid settings, we add another layer to the theoretical discussion on job demands and resources. Within the framework of the JD-R theory, leaders and leadership are primarily discussed as a potential resource or demand for employees (Bakker et al., 2023). Shifting the focus to leadership responsibilities as a job demand for supervisors reveals their bidirectional nature. Providing adequate leadership might be a resource for employees while constituting a demand for the supervisors because the associated tasks tie up their resources (Dóci et al., 2020; Stein et al., 2020; Zwingmann et al., 2016). However, in line with the proposed gain spirals in COR and JD-R theory (Bakker et al., 2023; Hobfoll et al., 2018), we assume that over time well-designed leadership tasks can also be a resource that feed back into the supervisors’ personal and conditional resources. In general, the work design perspective so far has been insufficiently considered in leadership research. However, as our results show, it is becoming increasingly important, especially in hybrid contexts. The development of an integrative leadership theory that encompasses the various mechanisms of leadership would enable a more holistic research approach.

### 5.2 Implications for future research and practice

Our study on leaders’ work characteristics in hybrid work settings highlights the importance of work design for leaders. Future research should investigate the interplay of (hybrid) work characteristics and leadership demands in relation to outcome variables like health, motivation or performance. For instance, our results suggest that hybrid work characteristics are likely to have a profile of job demands and resources that might differ from non-hybrid settings. We agree with Parker and Grote (2022) that testing the impact of a certain constellation of job demands and resources might deliver important insights regarding the optimal work design for different work arrangements. Here, it would be important to operationalize the precise arrangements of hybrid work accordingly (e.g., spatial/temporal distance, location; Hill et al., 2022). Additionally, time aspects could be considered by conducting longitudinal studies testing the gain or loss cycles that have been theoretically proposed (Bakker et al., 2023; Hobfoll et al., 2018; McClean et al., 2019).

Moreover, our research shifts the focus from leadership as a resource or demand for employees to its significance as a job demand for supervisors. This new perspective raises many questions, such as for instance how explicit leadership demands are spelled out, how conscious supervisors are about their tasks or what are the consequences for supervisors and employees when neglecting them.

Addressing these questions could help to identify specific tasks and competencies associated with hybrid leadership that could be incorporated in leadership development programs to provide supervisors with adequate development possibilities. To tame the chimera of hybrid work in practice, an in-depth analysis of the actual work characteristics of supervisors and employees—specifically, for the different locations and configurations that constitute hybrid work—might provide a good basis for adjusting job demands and resources accordingly. However, based on our results, we would recommend including organizational context factors like change processes and demands associated with leadership (e.g., on work design). Building on such an analysis, organizations could provide job resources that are tailored to the needs of their supervisors. Additionally, our findings indicate that organizations should offer a clear orientation on expectations and responsibilities regarding the role and tasks of supervisors in hybrid work settings to reduce ambiguity and insecurities.

### 5.3 Limitations

Our study is based on data obtained in expert interviews with 33 leaders. Even though the insights stem from a range of occupational backgrounds and demographics, future studies are needed to quantify and generalize the findings. In addition, the readiness to participate in a study on hybrid work might have led to a self-selection of supervisors that are, for example, particularly interested in the topic or have rather positive experiences. Furthermore, despite the semi-structured interview guidelines, the interviews were conducted by four different interviewers with personal differences in interview styles and focus that might have influenced answers of the participants.

For the deductive classification, we used the job demands and resources as categorized by the World Health Organization (2022) respectively by Dettmers and Krause (2020) to get a comprehensive overview of the hybrid work characteristics. Nevertheless, there are work characteristics based on other systematizations that are not covered in this classification. In addition to the theoretical problems, we discussed above regarding appraisal, the valence of the audio-recordings and the transcripts was not always clearly determinable. Multiple codes could be assigned to one text segment that additionally could be of differing length. For that reason, we decided not to report the number of codes for each category but still weighted the statements in terms of content based on their prominence across the interviews. That means, for instance, that some aspects were reported by almost all supervisors whereas others were only named by a few. Similarly, clearly assigning the leadership demands along the theoretical mechanisms of work design, direct interaction, and role modeling based on the statements was not always possible because they linguistically overlapped. Furthermore, the interview material is extensive, and we limited our scope to two research questions that we applied to the text. Thus, the focus is on work characteristics and leadership demands not taking personal variables on demographics or occupational background factors into account.

## 6 Conclusion

Our comprehensive analysis of supervisors’ work characteristics in hybrid work settings indicates a pattern of job demands and resources that might differ from exclusively onsite work. Moreover, hybrid work is associated with specific leadership demands that underline the importance of work design by supervisors. Thus, future research should investigate the special features of onsite versus off-site work and their interaction. For practice, we recommend an assessment of the work characteristics and leadership demands to enable adequate work design.

## Data Availability

The original contributions presented in the study are included in the article/Supplementary material, further inquiries can be directed to the corresponding author.
