# Supplementary material for: Taming the chimera of hybrid work: a work design perspective on supervisors’ working characteristics and leadership demands in hybrid work settings
Source: Front Psychol. 2025 Nov 12;16:1650717. doi: 10.3389/fpsyg.2025.1650717 (PMC12647059; doi:10.3389/fpsyg.2025.1650717)
Supplement: Supplementary file 1 [file Table_1.docx]

**Supplementary material**

| **Appendix A:**  *Demographics of the interviewees* | | | | | | |
| --- | --- | --- | --- | --- | --- | --- |
| **Code** | **Sex** | **Age (yrs)** | **Branch** | **Position** | **Tenure* current position** | **Tenure* overall as supervisor** |
| A1 | M | 28 | Brokerage | Team lead | 6 | 48 |
| A2 | F | 63 | Politics | Head officer | 144 | 240 |
| A3 | M | 36 | Engineering | Team lead | 90 | 138 |
| A4 | M | 43 | Education | Principal | 96 | 156 |
| A5 | M | 45 | Automotive | Team lead | 36 | 156 |
| A6 | F | 39 | Environmental protection | Team lead | 8 | 8 |
| B1 | F | 35 | Steel industry | Head of work council | 48 | 48 |
| B2 | M | 39 | Steel industry | Team lead | 96 | 96 |
| B3 | F | 41 | Service | Branch manager | 216 | 216 |
| B4 | M | 53 | Steel industry | Team lead | 84 | 84 |
| B5 | M | 41 | Steel industry | Team lead | 72 | 156 |
| B6 | M | 43 | Brokerage | Head of trade | 3 | 84 |
| B7 | F | 30 | Brokerage | Head of marketing | 18 | 18 |
| B8 | M | 36 | Brokerage | Head of controling | 48 | 69 |
| B9 | M | 57 | Brokerage | Head of consulting | 96 | 240 |
| B10 | F | 35 | Brokerage | Head of board | 90 | 120 |
| C1 | M | 38 | HR consulting | Senior consultant | 96 | 132 |
| C2 | F | 53 | Media | Team lead | 78 | 78 |
| C3 | F | 32 | Health care | Team lead, HR | 7 | 26 |
| C4 | F | 32 | HR consulting | Head of office management | 54 | 54 |
| C5 | M | 35 | Health care | Head of finance | 30 | 54 |
| C6 | F | 39 | Personnel consulting | Senior project management | 72 | 72 |
| C7 | F | 27 | Accounting | Team lead | 30 | 30 |
| C8 | F | 44 | Health care, R&D | Head of user research | 36 | 36 |
| D1 | F | 24 | Sales/ trade Business | Head of training | 2 | 2 |
| D2 | F | 49 | Sales/ trade Business | Team lead | 10 | 96 |
| D3 | M | 49 | Sales/ trade Business | Team lead, marketing | 24 | 300 |
| D4 | M | 40 | Sales/ trade Business | Sales manager | 48 | 96 |
| D5 | F | 36 | Sales/ trade Business | Head of department | 36 | 24 |
| D6 | F | 39 | Sales/ trade Business | Head of legal division | 6 | 48 |
| D7 | F | 54 | Sales/ trade Business | Head of HR | 240 | n/a |
| D8 | M | 40 | Sales/ trade Business | Head of operations | 15 | n/a |
| D9 | M | 38 | Sales/ trade Business | Division manager | 18 | 108 |

*Note.* F = female, M = male; age in years; *tenure in month; n/a = no data provided.

**Appendix B:** *Interview guidelines*

**Opening:**

- Warming up
- Standardized introduction of the topic

"As a supervisor, you play a key role in shaping the work of your employees. However, there is a need for research into the specific framework conditions that enable supervisors to implement this task appropriately. In order to optimize the work of your employees, it is necessary to understand the challenges, obstacles but also the resources of supervisors like you and to take a closer look at your personal working conditions. We are particularly interested in how you as a supervisor experience **hybrid working**. The term hybrid work refers to a mixture in which you and your employees work both onside and/or remote/ from home at times. This presents you as a supervisor with the challenge of not seeing everyone from your team onside every day, potentially expanding your own work but also your area of responsibility to remote settings.”

- Explanation of the structure/ proceeding of the interview
- Open questions of the interviewee?
- Demographics (age, sector and function, tenure as supervisor in the current position/ in general, number of employees supervised
- Start of audio recording -

**General questions regarding hybrid work:**

Leading question: What are the general framework conditions for supervisors in hybrid work settings?

**"You have now been able to gain some experience in working hybrid. How do you personally perceive the situation?"**

Potential follow-up questions:

- - *"How do you deal with the situation”*
  - *"How exactly does your hybrid working situation look like (onside, remote, commuting etc.)?"*
  - *"Can you give a specific example of this?"*
  - *"How long has the home office model been in place at your company? What timeframe are we talking about here?"*

***"What feedback do you receive from your employees regarding their hybrid situation? And how do you think about it?"***

- - *"Can you give an example?”*

**Questions about the understanding of the own role as supervisor:**

Leading question: How do supervisors in hybrid settings perceive their role (potential specific question regarding health)?

**"How would you describe your role as a supervisor in hybrid settings?"**

Potential follow-up questions:

- - *"Can you give an example (e.g. specific leadership tasks)?“*
  - *“How would you describe your leadership tasks in hybrid settings?”*
  - *"If you didn't work as a hybrid before, how has your role changed?”*

**"Hybrid working comes with new challenges (e.g. for health). How do you deal with this in relation to yourself and your team?"**

Potential follow-up questions:

- - *"Can you give an example?“*
  - *"How do you deal with your own health in this regard?"*
  - *"How do you deal with the health of your employees?"*
  - *"As a manager, you act as a role model for your employees. How would they describe your behaviour (e.g. in terms of care for health)?"*

**"How have you been prepared for your tasks as a supervisor, particularly with regard to the circumstances of hybrid working?"**

- - *"Can you give me a specific example of this?"*

**Hybrid work conditions:**

Leading question: What are the demands and resources of supervisors in hybrid work settings?

Transition to work conditions (provide examples/orientation: work content, work organization, working hours, social relationships, work environment)

**"What working conditions do you see as challenges, disadvantages or obstacles to hybrid leadership for you personally in your role as supervisor?"**

- - *"Can you give an example”?*

**"What working conditions do you see as advantages or resources for hybrid leadership for you personally in your role as supervisor?"**

- - *"Can you give an example”?*

**“To what extent do you have an influence on your personal work conditions in hybrid work?"**

- - *“Can you give an example of a work condition you were able to shape?”*
  - *“Can you give an example of a work condition that you were not able to shape, but that you would like to change?”*

**“To what extent do you have an influence on the work conditions of your employees in hybrid work?"**

- - *“Can you provide an example of a work condition you were able to shape?”*
  - *“Can you give an example of a work condition that you were not able to shape for your employees, but that you would like to change?”*

***„How do you experience the support of your own direct supervisor?“***

- - “Can you give an example*?*“

**Ranking task:**

Explain and conduct interactive ranking task

**Closing:**

- Thanks /Further procedure and contact
- *„How do you feel after the interview?“*
- *“Are there any open questions or comments you want to add?”*


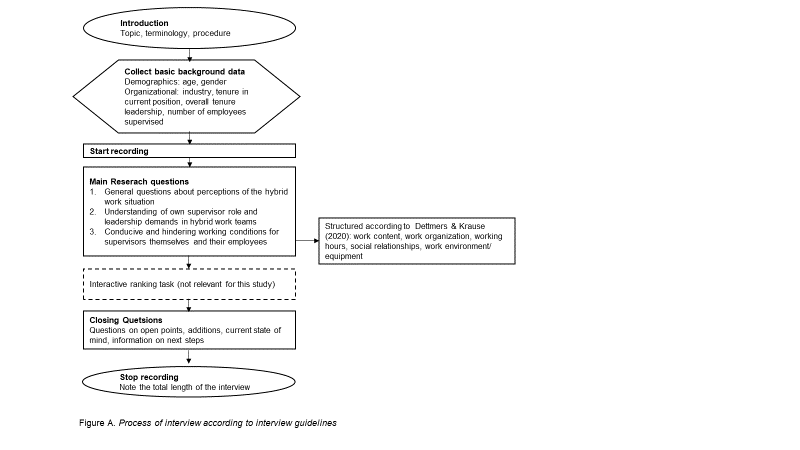


**Appendix C:** *Anchor examples of the German original and the English translation for hybrid working conditions of leaders*

| **Working condition** | **Code** | **Anchor example: German original** | **Anchor example: English translation** |
| --- | --- | --- | --- |
| **Work content** | | |  |
| Completeness of work tasks | Not coded |  |  |
| Autonomy* | C4_LG_43 | Gut für mich persönlich Flexibilität, ganz klar. Und man kann natürlich auch ein bisschen Sachen verändern, also auf andere Art und Weise verändern im Team. Wenn es jetzt in diesen Switch geht ins hybride Arbeiten, im Homeoffice, dass man einfach auch Möglichkeiten hat das anders zu gestalten. | Flexibility is good for me personally, of course. And of course, one can also change things a bit, change things in a different way in the team. When it comes to this switch to hybrid working, working from home, one simply has the opportunity to organize things differently. |
| Variability | C3_LG_39 | Also abwechslungsreich definitiv, einfach weil wir die Personalbetreuung von A bis Z machen. Da gibt es keine Langeweile, weil irgendwie nichts…, also klar einen Kern gibt es schon aber trotzdem ist vieles anders, also sehr divers. | So, it's definitely varied, simply because we do HR support from A to Z. There's no boredom, because somehow nothing…, so of course there's a core, but still a lot is different, so very diverse. |
| Emotional demands | B6_SDG_43 | Was mich allerdings belastet, das ist tatsächlich die eigene Verantwortung der Mitarbeiter gegenüber. Nicht, dass ich da nachts nicht schlafen kann, aber ich nehme auch viele Dinge mit nach Hause. Ich überlege mir ja auch, Mensch, wie geht es demjenigen jetzt, wieso hat er jetzt so und so reagiert, hast du dich vielleicht falsch ausgedrückt, hast du ihm oder ihr auf die Füße getreten. Wenn zum Beispiel Dinge an mich herangetreten werden, weil ich mir ein Vertrauensverhältnis aufgebaut habe. Und das beschäftigt mich. Und das belastet mich. Das ist aber denke ich mal auch Aufgabe. | However, what really weighs on me is my own responsibility towards my employees. Not that I can't sleep at night, but I also take a lot of things home with me. I also wonder how is this person doing now, why did he or she react this way or that, did I perhaps express myself incorrectly, did I step on his or her toes? For example, when things are said to me because I have built up a relationship of trust. And that bothers me. And that burdens me. But I think that's also my job. |
| Information overload* | D6_EA_179 | Wir haben finde ich einfach viel zu viele Kanäle inflow Kanäle. Du wirst ständig angezettelt auf Teams. Ich finds mega nervig. Du kriegst super viele E-Mails und dann halt vielleicht noch sogar mal ne WhatsApp oder halt dass jemand in der Tür steht und also gerade dieses Teams Chat zusätzlich zu Outlook empfinde ich als unangenehm muss ich sagen, weil du immer - und das kennst du, kannst dir ja psychologisch auch bewerten- du wirst ja immer wieder rausgerissen aus deinen Aufgaben. Du brauchst immer wieder Zeit. Also es ist ineffizient. Es ist nicht produktiv. | I just think we have far too many inflow channels. You're constantly being contacted at Teams. I find it really annoying. One gets a lot of emails and then maybe even a WhatsApp or someone standing in the door and I have to say that I find this Teams Chat in addition to Outlook unpleasant because one is always - and I mean that psychologically- I am always being pulled out of my tasks. You always need time. So, it's inefficient. It's not productive. |
| Lack of information* | B2_SDG_55 | Weil es ist, es geht auch immer damit einher, dass man halt ein gewisses Informationsdefizit aufbaut. Gerade, wenn ich jetzt sage, ich arbeite normal, ich arbeite immer so im Wechsel einen Tag zu Hause einen Tag hier, dann geht das. Aber, wenn wir jetzt mal zwei, drei Tage nicht vor Ort ist, ist es schon, glaub ich, könnte das relevant werden, dieses Informationsdefizit was man sukzessive aufbaut, wenn man halt auch nicht diesen Flurfunk nicht so mitbekommt, ne? | Because it always goes hand in hand with the fact that you build up a certain information deficit. Especially if I now say that I work normally, I always alternate between one day at home and one day here, then this works. But if we're not on site for two or three days, I think it could become relevant, this information deficit that gradually builds up, if you don't get to hear about it on the floor, right? |
| Qualification problems | C4_LG_67 | Es fehlt mir tatsächlich ein Mentor würde ich sagen. Also eine Person, die das so richtig gelernt hat, die Berufserfahrung in dem Bereich hat, die einem was beibringt. Das meiste ist halt schon learning by doing und sich ja durchfragen, durchlesen. Da fehlt tatsächlich noch eine Menge, was wünschenswert wäre da jemanden an die Hand zu bekommen oder zu haben. | I would say I really need a mentor. Someone who has really learned the ropes, who has professional experience in the field and who can teach you something. Most of it is just learning by doing and asking questions, reading through. There's still a lot missing, which would be desirable to have someone on hand. |
| Underutilization of skills | B7_SDG_55 | Ja tatsächlich. Viele Dinge, die mich unterfordern, weil das Team aktuell sehr schwach besetzt ist. Eigentlich besteht das Marketing aus zwei Leuten, mir und einer Vollzeitkraft. Die andere Kollegin ist Grafikern, die dann entsprechend andere Aufgaben hat. Dann muss man dann halt schon Aufgaben machen, die die Ameisen betreffen. | There are a lot of things that don't really challenge me because the team is currently very understaffed. The marketing team actually consists of two people, myself and one full-time employee. The other colleague is a graphic designer, who then has other tasks. Then you have to do tasks that usually is done by the “ants”. |
| **Work organization** | | |  |
| Role ambiguity* | B4_SDG_83 | Na ja, es ist es ist ein Spagat, ich hab quasi die höchstmögliche Flexibilität, bei schwammigen Vorgaben. Ja, was ich eben sagte. Wir haben grundsätzlich hier im Werk, die Pflicht, wer Homeoffice machen kann, soll das machen und am besten so viel wie möglich, um die Risiken einzudämmen. Gleichzeitig soll ich aber auch anwesend sein und ich müsste weniger anwesend sein. | Well, it's a balancing act, I have the greatest possible flexibility, so to speak, with vague specifications. Yes, what I just said. We basically have an obligation here at the plant that anyone who can work from home should do so and preferably as much as possible to limit the risks. At the same time, I should also be present, and I should be present less. |
| Work interruptions/ multitasking* | C8_LG_41 | Insofern ist das Slack schon eine ganz gute Abbildung der Büroarbeit. Man wird halt dauernd mit irgendwelchen Fragen oder so zumindest ganz kurz abgelenkt und muss dann wieder reinkommen und dann überlegt man sich noch innerlich, ja soll ich jetzt gleich antworten oder später und dann hat man es eben doch im Kopf. | In this respect, Slack is a pretty good representation of office work. You're constantly distracted with questions or something, at least for a short time, and then you have to come back in and then you're still thinking internally, yes, should I answer right now or later, and then you have it in your head. |
| Work intensity* | C4_LG_75 | Also ein ganz klares Zeitproblem ist, dass ich durch meine vielen Funktionen in sehr vielen Meetings bin. Also ich bin eigentlich die halbe Woche in Meetings kann man sagen, so round about je nach Woche was anfällt. Ich nehme aus fast jedem Meeting irgendwelche To-Dos mit, die sich dann bei mir stapeln. Wenn ich jetzt an die Auswirkungen auf mein Team denke, dadurch dass ich so viel in Meetings bin, bin ich dann halt auch nicht erreichbar. Das heißt, dass Personen auf ein Feedback von mir warten, nicht weiterarbeiten können. Ja das ist halt schwierig, gerade in der Einarbeitung, gerade in der Ausbildungssituationen. Wenn ich vier Stunden im Meeting bin, und alle warten auf ein Feedback von mir und dann kommt man raus und hat 36 E-Mails, die am besten jetzt sofort noch beantwortet werden müssen. | So, a very clear time problem is that I'm in a lot of meetings due to my many functions. I'm actually in meetings half the week, you could say, depending on the week. I take some to-dos with me from almost every meeting, which then pile up. When I think about the impact on my team, the fact that I'm in so many meetings means that I'm not available. This means that people are waiting for feedback from me and can't continue working. Yes, that's difficult, especially during familarization periods, especially in training situations. If I'm in a meeting for four hours and everyone is waiting for feedback from me and then you come out and have 36 emails that need to be answered right away. |
| Lack of communication* | A5_SK_64 | Dadurch verliere ich ganz konkret den Kontakt zu interdisziplinäre Abteilungen. Das heißt ich hab immer mit denselben Ansprechpartnerinnen und Kolleginnen zu tun, mit denen ich ähnliche Themen immer wieder bearbeite, bereite. Aber die, die außerhalb dieses Dunstkreises und Einflussbereiches, sondern Themenbereiche sind, die krieg ich gar nicht mehr mit. Also es gibt Mitarbeiterinnen in unserem Unternehmen, die habe ich seit zwei, zweieinhalb Jahren nicht gesprochen, nicht gesehen. | As a result, I lose contact with interdisciplinary departments. This means that I always have to deal with the same contacts and colleagues with whom I work on similar topics again and again. But I don't even notice the ones that are outside this circle and sphere of influence. So, there are employees in our company that I haven't spoken to or seen for two or two and a half years. |
| **Working time** | |  |  |
| Lack of time for recovery* | B5_SDG_50 | Zu Hause mache ich wenig Pausen bis gar keine Pausen. Hier, also wenn ich auf Arbeit bin, treffe ich mich regelmäßig, also nein, also wir gehen jeden Tag dann Essen. Das hab ich zu Hause nicht, entweder esse ich dann gar nicht oder man hat nicht wirklich so eine Hygiene. Man tippt dann noch irgendwie eine schnelle E-Mail beim Essen oder telefoniert also (…) Das habe ich persönlich nicht so gut im Griff, so will ich es einmal formulieren. | At home, I take little to no breaks. Here, when I'm at work, I meet up regularly, so no, we go out to eat every day. I don't have that at home, either I don't eat at all or I don't really have that kind of hygiene. Then I somehow type a quick email while eating or talking on the phone, so (...) I personally don't have a good grip on that, let me put it that way. |
| Work hour design | B2_SDG_83 | Dann war in der Regel Sonntags die Reparaturschicht und im Prinzip hatte ich mein Firmenhandy, ja,… mit wenigen Ausnahmen, relativ umfänglich am Mann, ja. Also eigentlich war ich immer erreichbar… Ähm, ja…. Gehört dazu. | Then there was usually the repair shift on Sundays and, in principle, I had my company cell phone, yes,... with a few exceptions, relatively extensively at hand, yes. So I was actually always available... Um, yes.... Is part of it. |
| Overtime* | C7_LG_21 | Also meine Überstunden sind doch auch weit in die Höhe geschossen. Da musste ich mich dann zwischendurch dann auch immer mal zurücknehmen und dann einfach mal sagen nein, diese Woche machen wir jetzt tatsächlich da auch mal einen harten Schnitt. Auch wenn auch ich das Gefühl hatte, ich könnte locker noch irgendwie vier Stunden weiterarbeiten, dann auch mal gesagt habe nein, nach acht Stunden ist jetzt hier dann eigentlich auch mal Ende. Aber auch da hatte ich das Gefühl, dass das auch in dem im Kreis der Führungskräfte sag ich mal auch immer weiter verschwommen ist. | My overtime has also skyrocketed. I had to take a step back from time to time and just say no, this week we're going to take a hard cut. Even though I had the feeling that I could easily continue working for another four hours, I sometimes said no, after eight hours it's actually over. But even then, I had the feeling that this was becoming increasingly blurred in the management circle, I would say. |
| Predictability* | C1_LG_27 | Dadurch dass man sich nicht mehr so oft sieht oder einfach nicht der klassische, also diese vielen Meeting-Points im Büro hat, ist mein Tag strukturierter geworden. | My day has become more structured because one does no longer see each other so often or simply does not have the classic meeting points in the office. |
| **Social relationships** | | |  |
| Social support | B8_SDG_65 | Mein persönlicher Anspruch ist, als Leiter die Angelegenheiten so zu regeln, wie ich das muss, aber wenn da mal eine Situation ist, die ich nicht klären kann, weil Informationen fehlen oder etwas eine Größenordnung hat, wo ich nicht mehr allein entscheiden kann oder sollte, weiß ich immer, wo die Türe ist, und da bekomme ich immer Rat und Unterstützung. | My personal aspiration as a leader is to manage matters as I have to, but if there is a situation that I can't resolve because information is missing or something is on a scale where I can't or shouldn't decide on my own, I always know where the door is and I always get advice and support. |
| Social stress | Not coded |  |  |
| Feedback and recognition | A6_SK_37 | Es gibt eine andere Teamleiterin hier bei uns. Die ist schon sehr lange Teamleiterin, sehr erfahren und da hab ich auch so einen kollegialen Austausch mit ihr, das ich alle paar Wochen irgendwie sende und so ein paar Punkte mitbringe wo sie auch eine Rückmeldung dazu gibt. Wie sie das einschätzt, ist zum Beispiel auch total hilfreich für mich. | There is another team leader here with us. She has been a team leader for a very long time, is very experienced and I also have a collegial exchange with her that I send every few weeks and bring back a few points where she also gives feedback. How she assesses it, for example, is also helpful for me. |
| **Work equipment & work environment** | | |  |
| Work equipment* | B2_SDG_7 | Es war für uns erstmal ein bisschen schwierig, überhaupt die Rahmenbedingungen zu schaffen. Also innerhalb der Abteilung, dass die Kolleginnen und Kollegen da entsprechende Endgeräte bekommen haben, um überhaupt ihre Arbeit aus der Ferne verrichten zu können. Dadurch, dass das ein Konstruktionsbereich war, war …, ja war die Anforderung nochmal ein bisschen höher, weil da halt auch nicht jeder Laptop genutzt werden kann, um jetzt zu konstruieren. | It was a bit difficult for us to create the framework conditions in the first place. In other words, within the department, ensuring that colleagues were given the appropriate devices to be able to work remotely. The fact that it was a design department meant that ..., yes, the requirement was a bit higher because not every laptop can be used to design. |
| Work environment* | B3_SDG_25 | Man hat nicht mal einen richtigen Bürostuhl, das hab ich zu Hause nicht. Das ist so ein ganz normaler Stuhl, so vom Esszimmer so ein Stuhl, da merkt man dann natürlich die Rückenprobleme. Also, das ist dieser ergonomische Arbeitsplatz, wo einfach der Arbeitgeber am Arbeitsplatz natürlich mehr Wert drauf legt, ein ordentlicher Schreibtisch, ein ordentlicher Stuhl, ausreichend große Bildschirme auch dass man das sieht, das ist zu Hause alles nicht so gegeben. | You don't even have a proper office chair, I don't have one at home. It's just a normal chair, a chair like from the dining room, so of course you notice the back problems. So, it's this ergonomic workplace, where the employer naturally places more value on having a proper desk, a proper chair, sufficiently large screens so that you can see it, and that's not the case at home. |
| **Organizational factors (inductive category)** | | |  |
| Organizational support* | C8_LG_21 | Wir haben einen begleitendes Führungskräfte-Coaching, das uns ermöglicht, ich glaube das ist immer alle zwei Monate oder alle sechs Wochen. Da gibt es immer so eine Session, wo wir unterschiedliche Themen bearbeiten und die sind oft auch frei. Also da geht es über Kommunikation oder über schwierige Themen ansprechen, auch potenziell über so hybride Themen. | We have an accompanying management coaching program that enables us to do this, I think every two months or every six weeks. There is always a session where we work on different topics, and they are often free. It's about communication or addressing difficult topics, potentially also hybrid topics. |
| Organizational culture* | A1_SK_99 | Das war ein harter Kampf, jetzt übers letzte halbe Jahr. Das wurde in unseren Führungsgremium häufiger diskutiert. Einfach weil wir den Sinn nicht verstehen. Es hat geklappt im Home Office, warum muss ich jetzt auf Zwang alle jetzt wieder ins Büro bringen? […]Und das ist, so diese Verbissenheit, manchmal ist das so ein zäher Kampf bis dann flächendeckend wirklich alle Führungskräfte auch sagen, ja doch, warum machen wir das denn nicht, klappt doch? | It's been a tough battle over the last six months. This has been discussed frequently in our management committee. Simply because we don't understand the point. The home office worked, why do I have to force everyone back into the office now? […] And that is, this doggedness, sometimes it is such a tough battle until all leaders across the board say, yes, why don't we do it, it works? |
| Organizational change | D7_EA_294 | Ne, Leute kommen nicht mit, ja, und die haben auch alle vor, bevor sie mit Transformation beschäftigt waren schon was zu tun gehabt, ja, und viele Mitarbeiter im Haus sind auch denke ich einfach mal verunsichert, weil des jetzt ja schon seit drei Jahren so geht, dass immer wieder ein anderes Thema hochkommt, ein anderes Programm kommt und irgendwann ist dann halt doch einfach mal ja der Zeitpunkt wahrscheinlich gekommen, wo man sich fragen muss, wie lange geht das jetzt noch so? Wann fängt hier mal wieder ein normales Arbeitsleben an? | No, people don't follow. Yes, and they all had something to do before they were involved with transformation, yes, and I think many employees in the company are simply unsettled, because it's been going on for three years now, that another topic keeps coming up, another program comes up and at some point, the time has probably just come where you have to ask yourself: “How much longer will this go on? When will normal working life start again?” |

*Note.* * Categories with direct references to hybrid work.

**Table Y.** Anchor examples of the German original and the English translation for hybrid leadership demands

| **Leadership task** | **Code** | **Anchor example: German original** | **Anchor example: English translation** |
| --- | --- | --- | --- |
| **Work design** | | |  |
| **Work content** | |  |  |
| Providing autonomy | C4_LG_41 | Virtuelles Arbeiten, bietet den Mitarbeitern natürlich mehr Autonomie und das sehe ich als klaren Vorteil. Wenn man halt eingearbeitet ist und dann relativ selbstständig läuft. | Virtual working naturally offers employees more autonomy, and I see that as a clear advantage. Once you've been trained, you can work relatively independently.” |
| Information management | C2_LG_39 | Manchmal sind es mir zu viele Kanäle, weil wir uns dann nicht fokussieren. Das ist auch immer wieder Thema in den Team- Routinen, in den großen Team-Routinen, wo wir sagen, also Freunde, lasst uns einfach nur einen Infokanal machen und dann liest man das, wenn man Zeit hat was zu lesen. | Sometimes there are too many channels for me because we can't focus. This is also a recurring theme in the team routines, in the big team routines, where we say, friends, let's just make an information channel and then you read it when you have time to read something. |
| Handling emotional demands | B1_SDG_23 | Und da muss man wahrscheinlich schon darauf achten, dass es nicht zu viel wird. Bezogen auf meine Mitarbeiter, speziell eine Mitarbeiterin, die ein... kleineres Kind noch hat… Glaube ich, dass es sehr schwierig ist von der Belastung, weil das Kind halt zu Hause ist und sie versucht nebenbei mobil zu arbeiten.. oder arbeitet auch mobil, also jetzt nicht, sie versucht es nicht.. (verlegenes Lachen) sie macht.. sie machst es. Und trotzdem ist ja das Kind immer irgendwie in der Nähe und möchte ja auch betreut werden. Und deswegen glaube ich, dass das eine besondere Herausforderung ist, das unter einen Hut zu kriegen und mit Sicherheit… stressiger, anstrengender als wenn man das hier am Schreibtisch macht und vielleicht ein bisschen mehr Ruhe hätte. | And you probably have to make sure that it doesn't become too much. With regard to my employees, especially one employee who has a...I think it's very difficult in terms of the workload because the child is at home and she tries to do mobile work on the side... or does mobile work, well not now, she doesn't try... (embarrassed laughter) she does... she does it. And yet the child is always around somehow and wants to be looked after. And that's why I think it's a particular challenge to get it all under one roof and certainly... more stressful, more exhausting than if you were doing it here at your desk and perhaps had a bit more peace and quiet. |
| **Work organization** | |  |  |
| Coordinating | B2_SDG_93 | Da fällt mir, ähm, der zusätzliche Workload den ich verteile und dann geht es glaub ich am Ende darum, wie intensiv bringe ich mich dann in die einzelnen Maßnahmen mit ein, wie übergebe ich solche Themen. Wie intensiv unterstütze ich solche Themen. Und das ist es natürlich eine Sache, die mir in Präsenz deutlich leichter fällt als jetzt im digitalen Raum. Also, mir selbst, persönlich fällt es leichter eine Stunde mit jemanden irgendwo hinzusetzen oder vor Ort was anzugucken, als ihm jetzt ein Outlet zu schreiben. | I have to deal with, um, the additional workload that I distribute and then I think in the end it's about how intensively I get involved in the individual measures, how I hand over such topics. How intensively do I support such topics? And that is of course something that is much easier for me in person than it is now in the digital space. So, for me personally, it's easier to sit down with someone somewhere for an hour or look at something on site than to write them an outlet. |
| Designing communication | B8_SDG_7 | Okay, ja im Grund genommen musste ich erst mal verstehen, dass es mehr darauf ankommt, ja die Kommunikation auch ein bisschen zu moderieren. Diese Gespräche, die wir sonst im Büro hatten, haben sich ja so nicht mehr ergeben und das war immer ein bewusster Anruf zu bewussten Problemen oder Fragestellungen und Anforderungen, die gerade da waren aber diese ganzen Zwischentöne und Zwischenebenen , die waren ja nicht mehr da, da hat man aufgelegt und war mit dem Thema durch. Das mussten wir irgendwie wieder einführen und herbeiführen auch und ähm, da hat es uns geholfen, dass wir einfach zwischendrin auch Teams anmachen, quasi im Hintergrund laufen lassen, jeder arbeitet , ist trotzdem Teams an, man kriegt so ein bissen mit, was der andere macht und wöchentliches Jour fix einfach auch, wo wir alle nochmal zusammensitzen und die ganze Woche Revue passiert wird und ja, mittlerweile haben wir das gut jetzt hinbekommen durch diese Mechanismen, die wir erarbeitet haben. | Okay, yes, basically I first had to understand that it was more important to moderate communication a little. These conversations that we used to have in the office no longer took place and it was always a deliberate call about deliberate problems or questions and requirements that were there at the time, but all these nuances and intermediate levels were no longer there. You hung up and were done with the topic. We had to somehow reintroduce that and bring it about and it helped us that we simply turn on Teams in between, let it run in the background, so to speak, everyone works, Teams is still on, you get a bit of an idea of what the others are doing and a weekly jour fix, where we all sit together again and review the whole week and yes, we have now managed to do that well thanks to these mechanisms that we have developed. |
| Providing role clarity | B5_SDG_5 | Wichtig ist, dass die Mitarbeiter wissen müssen, was ihre Aufgaben sind, was die Tätigkeiten sind, anderenfalls geht es nicht. Oder nicht wirklich gut, weil man doch für gewisse Dinge einfach dann vor Ort sein müsste, um das zu steuern. Also von daher, die Tätigkeit des Einzelnen muss bekannt und muss sicher auszuführen sein, das ist das A und O. | It is important that the employees know what their tasks are, what the activities are, otherwise it won't work. Or not really well, because you simply have to be on site for certain things in order to control them. So, from that point of view, the individual's job must be known and must be carried out safely, that's the alpha and omega. |
| **Working time** | |  |  |
| Designing working time | B7_SDG_17 | Eine Kollegin, sie ist Vollzeit, sehe ich häufig. Sie ist oft im Büro, weil sie sagt, sie kann zuhause nicht mehr gut arbeiten, möchte sie auch nicht mehr, hat sie anfangs während der Pandemie häufig gemacht, jetzt sagt sie möchte sie das nicht mehr. Die sieht man, wenn man öfter im Büro ist dann auch öfter. Ca. zwei bis dreimal die Woche, aktuell dann dreimal. Und die andere Kollegin, die ist auch Teilzeit, die ist immer einen Tag in der Woche da, da sind wir dann tatsächlich auch immer alle da. Also das versuchen wir dann schon einzurichten, also am Dienstag, dann gehen wir auch immer alle zusammen alles durch. Und an den restlichen Tagen ist sie dann im Homeoffice. Also an den restlichen zwei Tagen… die sehe ich dann einmal die Woche | I often see a colleague, she works full-time. She's often in the office because she says she can't work well at home anymore and doesn't want to, she did a lot at the beginning during the pandemic, but now she says she doesn't want to anymore. You see her more often if you're in the office more often. About two to three times a week, currently three times. And the other colleague, who is also part-time, is always there one day a week, so we're actually all there that day. We try to set that up, so on Tuesday, we always go through everything together. And then she works from home on the other days. On the other two days... I see her once a week. |
| Boundary management | C8_LG_17 | […] weil ich schon auch bei mir merke und auch bei meinen Mitarbeiterinnen merke, dass sie öfter mal auch dann länger arbeiten. Also man neigt dazu die Grenze nicht so zu ziehen. Da kann ich auch nur ermutigen zu sagen, das ist in Ordnung, bitte teilt euch die Arbeit so ein, dass ihr das schafft in der Zeit und ansonsten muss das eben warten, gib mir einfach ein Feedback. | I've noticed that me and my employees often work longer hours. So, you do not tend to draw the line like that. I can only encourage them to say, that's okay, please organize your work so that you can manage it in the time available and otherwise it will just have to wait, just give me feedback. |
| Controling working time | C4_LG_29 | Bei der Auszubildenden sehr, sehr streng versuche ich da vorzugehen. Das heißt, dass sie Pausen einhält, dass sie Pausen auch regelmäßig macht, dass sie keine Überstunden macht. Wenn sie Überstunden macht, dass sie die auch innerhalb von zwei Wochen abbummelt, dass wir da immer auf einem Level sind. Das heißt, da kontrolliere ich auch tatsächlich die eingetragene Arbeitszeit, um das im Blick zu haben. Bei den anderen Mitarbeitern gucke ich auch regelmäßig rein, was die Arbeitszeiterfassung und wie das Überstundenkonto aussieht, um das im Blick zu haben, was einfach so von den Kapazitäten auch los ist, ob irgendwo eine Entlastung notwendig ist. Wir haben noch regelmäßige Meetings, wo wir über so was sprechen, so dass man einfach weiß, ok hier ist keiner der komplett ausgebrannt ist. | I try to be very, very strict with the trainee. That means that she takes breaks, that she takes regular breaks, that she doesn't work overtime. If she does work overtime, I make sure that she works it off within two weeks, that we are always at the same level. In other words, I check the recorded working hours to keep an eye on things. I also regularly check the other employees' working time records and overtime accounts to keep an eye on what's going on in terms of capacity and whether any relief is needed. We still have regular meetings where we talk about these things so that we know that nobody here is completely burnt out. |
| **Social relationships** | |  |  |
| Establishing social relationships | D1_EA_25 | Weil halt gerade das Onboarding von Auszubildenden und dualen Studenten in neuen Abteilungen aktuell deutlich schwieriger ist als persönlich. Teilweise trauen sich auch junge Menschen vielleicht nicht so sehr anzurufen, was zu schreiben, nachzufragen, wenn was unklar ist in den Abteilungen. Von dem her ist der Wunsch von allen eigentlich, dass es wieder mehr vor Ort ist und dann eben auch die Fachabteilungen mehr vor Ort sind und man dann einfach dadurch, sag ich mal näher zusammenarbeiten kann und die Motivation fällt den sowieso teilweise schwer. | Because the onboarding of trainees and dual students in new departments is currently much more difficult than in person. In some cases, young people are perhaps not so confident to call, write something or ask if something is unclear in the departments. In this respect, everyone would actually like to see more on-site work again and for the specialized departments to be more on-site, so that we can work together more closely and motivation is sometimes difficult anyway. |
| Facilitating social contact | C1_LG_29 | Gibt es Touchpoints um in dieses Thema Abstimmung, was ich vorher ja auch schon genannt hatte, jetzt gar nicht nur inhaltliche Abstimmungen zu Aufgaben, sondern eben auch Abstimmungen zu Fragen wie „wie geht es mir“, habe ich irgendwelche Themen, die ich anbringen möchte. Also da auch Touchpoint zu schaffen, das ist sicherlich eine Herausforderung von Führung. | There are touchpoints for this topic of coordination, which I mentioned earlier, not just coordination on the content of tasks, but also coordination on questions such as "how am I doing", do I have any issues that I would like to raise? So, creating touchpoints is certainly a leadership challenge. |
| Problem solving | B9_SDG_3 | Wir sind ja inzwischen in einer hybriden Situation, sodass die Mitarbeiter wieder drei Tage die Woche hier in Präsenz sind und dadurch verschieben wir die Konflikte oder die Lösungsgespräche auf die Präsenz. | We are now in a hybrid situation, which means that employees are back on site here three days a week and that we have to postpone conflicts or resolution discussions until we are on site. |
| Designing feedback | C6_LG_33 | Feedback zu bekommen, Feedback zu geben, uns gegenseitig Lob auszusprechen, weil wir sehr intensiv in unterschiedlichsten Konstellationen zusammen arbeiten, also beispielsweise durch eine monatliche Teambefragung, aber eben auch durch Mitarbeitergespräche, Feedbackgespräche, die insbesondere auch im Onboarding natürlich nochmal deutlich wichtiger geworden sind und uns auch nochmal standardisierter und strukturierter auch abgehalten werden. | Getting feedback, giving feedback, giving each other praise because we work together very intensively in a wide variety of constellations, for example through a monthly team survey, but also through employee appraisals, feedback meetings, which have of course become much more important, especially in onboarding, and are also held in a more standardized and structured way. |
| Staff-care | A1_SK_93 | Heißt also, in der Woche in der sie dann nicht diese, diesen Kinderzwang hat, das klingt jetzt wieder sehr, sehr böse an, aber diesen Zwang abzuholen, dass ist ja nun mal eine Pflicht in diesem Fall als Mutter beziehungsweise als Vater. In diesem Fall ist es eine Mutter, deswegen habe ich das so gesagt. Das zu umgehen und da haben wir gesagt, ach komm, die eine Woche ein bisschen mehr und dafür gebe ich dir die andere Woche Home Office. So, diese Person hätte sonst nur die Chance sich ein arbeitsnäheren Arbeitsplatz zu suchen, der diese Bedingungen erlaubt. Und da ich die Person nicht verlieren will, hat hier sich die Firma unheimlich stark geöffnet. … Heißt also in Summe: Ich habe eine Chance vielmehr auf Einzelschicksale einzugehen. | **In other words, in the week in which she doesn't have this, this obligation for** childcare, that sounds very, very nasty again, but **picking up** is an obligation in this case as a mother or as a father. In this case it's a mother, that's why I said it like that. To get around that**, we said, oh come on, a bit more one week and I'll give you the other week home office in return. So, this person would otherwise only have the chance to look for a job closer to work that allows these conditions. And since I don't want to lose this person, the company has accommodated her a lot. ... So, all in all, this means that I have a chance to respond to individual fates.** |
| **Work environment** | |  |  |
| Facilitating an adequate work environment/ equipment | D3_EA_29 | Schreibtischstühle oder so also jederzeit kann sich jeder auch das mit nach Hause nehmen oder auch den höhenverstellbaren Schreibtisch, wenn der zu Hause da reinpasst, also wer das haben möchte, dem hab ich das auch genehmigt und da hatten wir auch drei, vier Mitarbeiter, die haben uns dann ihren Bürostuhl mit nach Hause genommen. | Desk chairs or something like that, so anyone can take them home at any time, or even the height-adjustable desk, if it fits in at home, so anyone who wants that, I've also approved it and we've also had three or four employees who have then taken their office chairs home with them. |
| **Direct interaction** | | |  |
| Communication quality | A5_SK_9 | Naja, also im Face-to-Face Gespräch kannst du ganz anders reagieren und ganz anders Gefühle rüber bringen und so weiter. Und, wenn du das in Worten getippt machen willst irgendwie oder wie auch immer, dann führt das ganz oft zu Missverständnissen oder es braucht sehr viel Formulierungsarbeit irgendwie um Sachen wirklich so rüber zu bringen, wie man es auch sagen will, damit da keine Missverständnisse entstehen. Und manchmal sind Gespräche zwischen Leitungen und Kollegium auch manchmal Konfliktgespräche. Oder wo es um persönliche Probleme auch geht, wo man drauf eingehen muss und so weiter. Und das ist natürlich dann herausfordernd, dass irgendwie über Mail zu machen. | Well, in a face-to-face conversation you can react very differently and convey feelings very differently and so on. And if you want to type it out in words or whatever, it often leads to misunderstandings or it takes a lot of formulation work to really get things across the way you want to say them so that there are no misunderstandings. And sometimes conversations between management and staff are also sometimes conflict discussions. Or about personal problems that need to be addressed and so on. And of course it's challenging to do that somehow via email. |
| Perception of problems | D6_EA_25 | […] sind sie erschöpft? Sind sie müde? Sind sie gestresst? Sind sie nervös? Ja, wenn du jemandem auf dem Flur siehst oder im Büro nebenan, dann merkst du sowas. Wenn jemand einfach nicht da ist und du siehst sie nicht, dann merkst du das nicht unbedingt, weil die Leute sich dann auch bei einem Telefonat vielleicht zusammennehmen und nicht so offen damit umgehen. | […] are they exhausted? Are they tired? Are they stressed? Are they nervous? Yes, if you see someone in the corridor or in the office next door, you notice that. If someone just isn't there and you don't see them, then you don't necessarily notice it, because then people might also pull themselves together during a phone call and not be so open about it. |
| Immediate feedback | B1_SDG_49 | Also, ich kann natürlich (ähm)… Was wir jetzt gerade auch als Thema hatten, die (die) Kommunikation, da kann ich persönlich natürlich meinen Anteil zu geben. Ich kann sagen, „Hey super gemacht! Toll, klasse!“. Kann ich schriftlich machen, kann ich anrufen. Das kann ich alles machen. | So, of course I can (um)... What we just had as a topic, the communication, I can of course personally contribute to that. I can say, "Hey, great job! Great, great!". I can do that in writing, I can call. I can do all that. |
| Responsiveness | D3_EA_57 | Hinsichtlich Führung hinsichtlich Team zu sehen, man ist doch auch schneller da, also schneller ansprechbar, sag ich jetzt mal, weil es ist oftmals einfach nur ein Klick und du kannst sofort jemanden, du hast sofort jemanden dran oder Teams-Chat du. | In terms of leadership with regard to the team, you're also there faster, so you can be contacted faster, I say. Because it's often just a click and you can get someone immediately, you have someone on the line immediately or you can chat in Teams. |
| **Role modeling** | |  |  |
| Reflecting one’s role | B1_SDG_15 | Und das finde ich auch als Führungskraft auch schwierig, da nicht irgendwie in diese kontrollierende Rolle überzugehen. Weil ich finde, dass gehört sich nicht, das mache ich hier auch nicht. Aber trotzdem irgendwie zu versuchen, alles zusammenzuhalten und die Arbeiten dann auch erledigt zu wissen. | And as a manager, I also find it difficult not to take on this controling role. Because I don't think that's appropriate, and I don't do that here either. But I still somehow try to keep everything together and make sure the work gets done. |
| Recognizing own area of responsibility | C2_LG_19 | Ich achte aber auch auf die ersten; also mir ist es unglaublich wichtig, dass wir, also bei mir aus dem Team niemand mit einem Burnout nach Hause geht, weil da bin ich dann der die Erste, die bei [Name des Vorgesetzten] anklopft und sagt, so wir können nicht weitermachen mit der Arbeitsbelastung wie vorher mit zwölf Personen, | It's incredibly important to me that no one in my team goes home with burnout, because I'm the first one to knock on [supervisor's name]'s door and say we can't carry on with the same workload as before with twelve people. |
| Role modeling | C1_LG_39 | Aber alles was ganz klassisch zugeordnet werden kann, das erfasse ich in der Regel schon und das ist mir aber auch wichtig, eben zum einen um selbst langfristig ja belastbar zu bleiben und auch weil ich es eine schwierige Vorbildfunktion finde, wenn man eigentlich immer zeigt, es geht eigentlich nur, wenn man Überstunden macht. | But everything that can be classically assigned, I usually pick up and that is also important to me, on the one hand to remain resilient in the long term and because I find it difficult to set an example if I always show that I can only do it if I work overtime. |
| **Follower characteristics (inductive category)** | | |  |
| Follower characteristics | C5_LG_15 | Also allein, bis wir alle Mitarbeiter technisch und zugegeben auch intellektuell befähigt hatten, dass das überhaupt funktioniert, war schon ein relativ schwieriger Weg. Weil einfach die technische Affinität nun mal gerade bei älteren Kollegen nicht so hoch ist und auch die Akzeptanz teilweise. Was zum Beispiel so ein Problem ist, dass man sagt man ermöglicht das mobile Arbeiten, was aus meiner Sicht trotzdem irgendwie ein Entgegenkommen an den Mitarbeitern ist und verlangt dafür natürlich im Gegenzug das Umstellen des Telefons, Nutzung von Videokonferenzen usw. und diese Rückkopplung ist dann wiederum relativ schwierig. Man hört dann, das will ich nicht, ich habe kein Mikrofon und all diese Geschichten. Also das hatte ich bei einigen, dass das eher so ist, gerne alles nehmen an Flexibilität aber dann Geben im Sinne von ok, jetzt werden auch andere Dinge von mir verlangt, das ist dann halt schwierig. | So, it was a relatively difficult journey just to get all our employees technically and, admittedly, intellectually capable of making it work. Simply because the technical affinity is not so high, especially among older colleagues, and also the acceptance in some cases. For example, it's a problem that you say you're enabling mobile working, which in my view is somehow a concession to the employees, but in return, of course, you ask them to change their phones, use video conferencing etc. and this response is relatively difficult. You then hear, I don't want that, I don't have a microphone and all these stories. I've had that with some people, that it's more a case of taking everything in terms of flexibility but then giving it in the sense of ok, now other things are being asked of me, that's just difficult. |
| Performance | A3_SK_24 | Wir merken das auch, wir sind auch produktiver, definitiv. Es kommt am Ende mehr raus. Wenn ich jetzt so sage, wie lange brauchst du für die Anlage? Dann sagt er, ja zwei Tage. Da ist er einen Tag im Homeoffice, dass er sich sagt, du ich mach jetzt noch mal eben eine Stunde sauber mal hier und dann kann das Ding geprüft werden. Also da sieht man schon, die sind dann schon wesentlich schneller. | We notice that too, we are also more productive, definitely. We get more out of it in the end. If I say now, how long do you need for the system? Then he says, yes, two days. He's in the home office for a day, so he says, I'll just spend an hour cleaning up here and then the thing can be checked. So, you can see that they are much quicker. |

**Table Z.** Translation of further quotes

|  | **Code** | **Anchor example: German original** | **Anchor example: English translation** |
| --- | --- | --- | --- |
| **Working conditions** | | |  |
| **Work content** | |  |  |
| Less emotional demands (resource) | C6_LG_59 | Das ist natürlich etwas anderes, unangemessenes Verhalten ist natürlich in real live eine ganz andere Hausnummer als virtuell. Wenn ich mir jetzt überlege, was könnte unangemessenes Verhalten sein, also rein hypothetisch gesprochen irgendwer macht dich irgendwie doof an, also Kandidat oder Kunde macht dich irgendwie doof an, das ist jetzt halt virtuell im Video-Call nicht ansatzweise so unangenehm wie es halt face-to-face wäre. Also so richtig face-to-face. Da federt es das natürlich schon so ein bisschen. | Inappropriate behavior is a completely different matter in real life than virtually. If I now think about what inappropriate behavior could be, hypothetically speaking, someone is somehow making a fool of you, i.e. a candidate or customer is somehow making a fool of you, that's not nearly as unpleasant in a virtual video call as it would be face-to-face. So, really face-to-face. Of course, that cushions it a bit. |
| **Work organization** | |  |  |
| Less interruptions (resource) | B7_SDG_67 | Im Büro mittlerweile häufiger, wenn mehr Leute da sind, wird man auch häufiger unterbrochen. Homeoffice tatsächlich weniger im Vergleich, wenn man sich da aus der Telefonleitung auch rausnimmt und wenn man alles bedacht hat, wird man da nicht so häufig gestört. Ist tatsächlich besser, ja. | In the office, more often now [referring to interruptions], when there are more people there, you are also interrupted more often. Working from home is actually less so, in comparison, to the office, if you take yourself out of the phone line and if you’ve thought everything through, you’re not interrupted as often. It’s actually better, yes. |
| Better responsivity (resource) | A3_SK_5 | Und durch diese neuen Kommunikationsmöglichkeiten, wie Teams ist die Verbindung immer eins A zur Firma. Egal wer was hat, kann sich jederzeit melden, man kann immer unterstützen. Das klappt wirklich sehr gut. | And thanks to these new communication options, such as Teams, the connection is always perfect to the company. No matter who has something, you can always get in touch, you can always provide support. It works really well. |
| **Working time** | |  |  |
| Time gain (resource) | B2_SDG_55 | Ja, definitiv... also grundsätzlich ein Zeitgewinn. Dadurch, dass man, das ich mir die Fahrt spare. | Yes, definitely... So, basically a time saving. The fact that one... that I save myself the drive. |
| Increased flexibility (resource) | B7_SDG_13 | Also da bin ich total flexibel. Da pass ich mich auch dem an, was gerade so ansteht. | So, I'm totally flexible. I adapt to what's going on at the time |
| Life-domain-balance (resource) | B2_SDG_55 | Das ist einfach dieses Thema Work-Life-Balance was sicherlich auch als Führungskraft noch mal eine andere Herausforderung ist. Wenn man, … Es wird dann schon erwartet, dass man auch zeitlich einfach mit einer anderen Präsenz dabei ist. Das kann man, wenn man mobile arbeitet, ich sag jetzt mal, ein bisschen, ja, schöner gestalten, dass halt für einen selbst auch noch ein bisschen mehr Zeit übrig bleibt. Das ist für mich auf jeden Fall der größte Vorteil. | It's simply this issue of work-life balance, which is certainly another challenge as a manager. If you... It's expected that you're also available with a different attendance in terms of time. **If you work remotely, I'd say you can make it a bit, yes, nicer so that you have a bit more time left for yourself. That's definitely the biggest advantage for me.** |
| **Social relationships** | |  |  |
| Lack of feedback (demand) | C2_LG_5 | Erstmal, wir haben gesagt erstmal vier Wochen, wenn ich euch vielleicht auch ein halbes Jahr nicht sehe, und dann waren es plötzlich sehr, sehr viele Monate mehr, die man halt die Kollegen auch immer nur virtuell im Anschlag hatte und ja ich fand es wirklich am Anfang schwierig, wenn man so wenig Anleitung auch von seiner Führungskraft bekommen hat. | First of all, we said four weeks, if I don't see you for maybe half a year, and then it was suddenly many, many more months that you only ever had virtual contact with your colleagues and yes, I really found it difficult at the beginning **when you got so little guidance from your supervisor.** |
| Lack of recognition (demand) | A6_SK_53 | Das ist,... ich glaub, da ist so mehr Wertschätzung ein Mangel. Ich glaub, da ist so ein Gefühl von .. oder mehr ein Gedanke von, ey wir sind auch wichtig, warum habt ihr uns vergessen? | **I think there is a lack of appreciation.** I think there's a feeling of ... or more a thought of, ey, we're important too, **why do you have forgotten us**? |
| **Work environment** | |  |  |
| Adequate equipment (resource) | A5_SK_54 | Und das geht heutzutage natürlich aber auch deutlich besser als damals, weil mit Sharepoint, mit One-Drive, Dokumente eben geteilt werden können oder auch Bildschirme eher geteilt werden, als das früher der Fall war. Sondern da hat man sich irgendwelche Dokumente hin und hergeschickt sich zusammengerufen und sagte, auf Seite so und so Abschnitt so und so ja, das war dann schon deutlich komplizierter. Heutzutage geht das mit Teams wirklich sehr, sehr gut. Auch das gemeinsame Arbeiten in Dokumenten funktioniert deutlich besser als das noch vor einigen Jahren war also da, hat doch die Technologie einen großen Anteil daran, dass es jetzt heute so gut funktioniert. | And nowadays, of course, this is much better than it was back then, because **Sharepoint and OneDrive allow documents to be shared or screens to be shared more easily than was the case in the past. Instead, you used to send documents back and forth to each other and say, on page so and so section so and so, which was much more complicated.** Nowadays, Teams functions really, really well. Working together on documents also works much better than it did a few years ago, so technology has played a big part in the fact that it works so well today. |
| Adequate environment (resource) | D2_EA_69 | Also was halt extrem wichtig ist, ist dass man einfach einen guten Laptop, also gute Arbeitsmaterialien zur Verfügung gestellt bekommt und auf der anderen Seite, ich hab halt einen total lieben Mann und der hat mir halt das, das Büro hier komplett eingerichtet. Er hat also wirklich neuen Stuhl, eigentlich, ich habe hier echt diesen Riesenbildschirm. Das sind vier kleine Bildschirme in einem und ich arbeite super gerne damit. Das ist eher im Büro so, da habe ich ja die zwei Bildschirme nebeneinander stehen und dann noch einen Laptop. Das ist eher so hinderlich für mich. | So, what is extremely important is that you simply get a good laptop, i.e. good working materials, and on the other hand, I have a really lovely husband and he has completely furnished my office here. He really has a new chair, actually, I have this huge screen here. It's four small screens in one and I really like working with it. It's more that in the office, where I have the two screens next to each other and then a laptop. That's more of a hindrance for me. |
| **Organizational factors** | | |  |
| Lack of organizational support | A5_SK_54 | Nein, also es ist, es gab in dem Sinn im Vorfeld eben keine Schulungen oder Trainings, wie man einen Mitarbeiter auf Distanz führt, ne. | **No, it's just that there was no training in advance on how to lead an employee at a distance.** |
|  |  |  |  |
| **Leadership tasks** | | | |
| Work design | B7_SDG_37 | Das alles viel digitaler geworden ist und wir im Team mehr Struktur haben, weil wir gemerkt haben, also wir haben eine Arbeitsplattform, die nennt sich Wiki, das ist eigentlich so etwas wie Wikipedia, so ähnlich aufgebaut und da haben wir sehr viel in der Zeit, also in der ganzen Phase wirklich auch gelernt damit zu arbeiten und das digital zu machen und uns da zu strukturieren und das gibt mir als Führungskraft auch immer, da weiß ich, da kann ich gucken, wie weit was ist, da sind wir schon viel digitaler geworden. Das ist echt… Ja das ist ein großer Vorteil. Es bringt auch in der Führung viel mehr, wenn man weiß, wo die Sachen sind und man muss nicht anrufen und fragen und so weiter. Oder das sagt, hier auf der Seite guck mal… Also das ist schon viel viel einfacher als vorher. | Everything has become much more digital and we have more structure in the team because we have realized that we have a work platform called a wiki, which is actually something like Wikipedia, structured in a similar way, and **we have learned a lot during this time, i.e. during the whole phase, to work with it and to do it digitally and to structure ourselves there and that always gives me as a leader…, I know, I can see how far something is, we have already become much more digital. That's really... Yes, that's a big advantage. It's also much more useful in leadership if you know where things are and you don't have to call and ask and so on.** Or saying, look here on the site... So, it's much easier than before. |
|  | C8_LG_27 | Ja, dass es ein bisschen organisierter ist wie ich mit meinen Mitarbeitern umgehe. Also es gibt einfach sehr konkrete Wochenplanungsmeetings, es gibt ein Tool dafür, ein Werkzeug was das erleichtert. Es gibt auch bestimmte, wir haben als Company einen Loop-Approach, das heißt wir haben auch bestimmte Zeiten, wo wir uns als Team, im größeren Team, noch über bestimmte offene Fragen, also Governance Issues austauschen oder allgemein über so was wie Office Vibes, also wie geht es allen, darüber wird noch mal gesprochen, wie man es vielleicht verbessern kann. Also das sind schon alles gute Möglichkeiten, um ... einfach auf Distanz auch miteinander zu kommunizieren, zu kollaborieren. | **Yes, the way I deal with my employees is a bit more organized. So, there are simply very specific weekly planning meetings, there is a tool for this, a tool that makes it easier.** There are also certain, as a company we have a loop approach, which means we also have certain times when we as a team, in the larger team, discuss certain open questions, i.e. governance issues, or generally about something like office vibes, i.e. how everyone is doing, we talk again about how we can perhaps improve things. So, these are all good opportunities to ... **simply communicate and collaborate with each other at a distance.** |
| Work design | B7_SDG_25 | Also ich achte schon darauf, dass die Termine, wenn wir alle im Jourfix sind… oder das Jourfix haben, dass wir alle im Büro sind. Das ist mir schon wichtig, dass dann wirklich auch alle da sind, dass wir nicht immer vor den Bildschirmen hängen und vor allem, dass wenn wir noch andere Termine im Team selber haben, dass wir die auch auf den Tag verlegen. Da achte ich schon drauf und das ist mir auch wichtig, weil ich finde auch, face-to-face ist dann schon ein bisschen einfacher manchmal diese Themen zu klären. | **I make sure that when we're all in the jourfix... or have the jourfix, that we're all in the office.** It's important to me that everyone is really there, that we're not always glued to our screens and, above all, that if we have other appointments in the team, that we reschedule them for the day. I make sure of that and it's important to me, because I also think it's sometimes a bit easier to clarify these issues face-to-face |
| Work design | B6_SDG_25 | Aber auch Empathie, also zwischenmenschliche Dinge, die gehen aus meiner Sicht sehr stark verloren. | But empathy, i.e. interpersonal things, are also very much lost in my view. |
| Work design | B4_SDG_83 | Ich könnte besser führen von Fern, also auf Distanz das Führen machen, wenn ich, wenn die Mitarbeiter bessere Hardware hätten. Das geht aber nicht, weil ist nicht verfügbar. Ich kann nicht nur wegen der Pandemie jetzt komplette Rechnersysteme austauschen, damit sie ihre Kamera anschließen können. Also ist das tatsächlich eine echtes Hardwareproblem. Sachen, die funktionieren, wegzutun und durch was Höherwertiges zu ersetzen. Also da geht es auch ums Geld und um gegebenenfalls Sinnhaftigkeit. | **I could lead better from a distance, i.e. from a distance, if I, if the employees had better hardware. But that's not possible because it's not available.** I can't replace entire computer systems just because of the pandemic so that they can connect their camera. So, this is actually a real hardware problem. Getting rid of things that work and replacing them with something better. So, it's also a question of money and, if necessary, usefulness |
| Direct leadership | B2_SDG_61 | Ist im Prinzip sehr vergleichbar. Also wenn ich im Büro bin und er ist im Büro, dann ist es schon die Regel, dass er so zwei bis drei Mal am Tag bei mir im Büro steht oder ich bei ihm bin und man gewisse Dinge abstimmt. Und dann ist auch genau das mit diesem Feedback, was halt dann auch genau in solchen Situationen passiert. Bei der digitalen Kommunikation ist es dann halt schon, auch was anderes. Eine E-Mail hat nicht mal eben so schnell geschrieben, wie man mal eben den Kopf irgendwo reinsteckt oder man trifft sich an der Kaffeemaschine beim Kaffee holen. Das sind halt so Sachen, die beim mobilen Arbeiten auf der Strecke bleiben. | It's very similar in principle. So, when I'm in the office and he's in the office, it's the rule that he's in my office two or three times a day or I'm with him and we coordinate certain things. And then this feedback is exactly what happens in these situations. **With digital communication, it's a different story. An email isn't written as quickly as you just stick your head in somewhere or you meet at the coffee machine to get coffee.** These are things that are lost when it comes to mobile working. |
| Role modeling | A6_SK_53 | Manchmal brauche ich einen Moment für mich, wo ich mich zurückziehe und mir noch mal vergegenwärtige, was ist eigentlich das große Ganze? Was ist eigentlich das Dach davon? Was mach ich hier eigentlich? Weil sich das manchmal so vielfältig anfühlt, dass ich von der Angst her merke von, oh, verliere ich mich jetzt im Detail? Bin ich jetzt hier überhaupt noch auf dem richtigen Pfad? Was ist eigentlich der, worum geht es hier eigentlich? Ja was ist eigentlich (Tonaussetzer)? Passt das überhaupt noch zu unserer Strategie? Passt das überhaupt noch auf unser Ziel? Und da merke ich, dass ich dann schon ab und zu mal so einen Moment brauche, wo ich mir ganz schnell strategisch Gedanken mache. Und mich wieder einnorde. Auch vielleicht das Gespräch suche mit meiner Vorgesetzten, auch nochmal zu gucken, ob ich da auf dem richtigen Weg bin überhaupt? | Sometimes, I need a moment for myself to step back and think about what the big picture actually is. What is actually the roof of it? What am I actually doing here? Because sometimes it feels so multifaceted that my anxiety makes me realize, oh, am I getting lost in details now? Am I still on the right path here? What is actually the right path, what is this actually about? Yes, what is actually (audio dropout)? Does this still fit in with our strategy? Does it still fit our goal at all? And that's when I realize that every now and then I need a moment like this to think strategically very quickly. And get myself back on track. Maybe I also need to talk to my line manager to see if I'm on the right track? |
| Role modeling | B6_SDG_5 | Also ich nehme es als herausfordernd wahr, weil bestimmte Dinge nicht durch die Technik ersetzt werden können. Ich nehme mal das Beispiel Emotion, Spüren, Fühlen, die Mimik, die Gestik des Gegenübers, ist über einen Bildschirm deutlich schwieriger wahrzunehmen. Ich habe es anfänglich auch als unangenehm empfunden, weil ich sagen muss, dass mein mein Anspruch als Führungskraft ja genau derjenige ist, dass ich diese Antennen habe. Es war etwas wo wir uns, ich sag mal, alle mit lernen mussten umzugehen. Es ging nun mal nicht anders. Wir konnten die Menschen nicht alleine lassen. | So, I perceive it as challenging because certain things cannot be replaced by technology. Let me take the example of emotions, sensing, feeling, the facial expressions and gestures of the other person, which are much more difficult to perceive via a screen. I also found it uncomfortable at first, because I have to say that my claim as a leader is precisely that I have these antennae. It was something we all had to learn to deal with. There was no other way. We couldn't leave people on their own. |
| Role modeling | B1_SDG_51 | Es wird keiner angewiesen außerhalb der normalen Arbeitszeit zu arbeiten, auf gar keinen Fall. Das erwarte ich hier nicht, das erwarte ich auch zu Hause nicht. Aber wenn Sie das gerne machen möchten, weil es halt für sie grad irgendwelche Vorteile bringt, dann sollen sie das tun. Aber natürlich hat man dann weniger Einfluss darauf, ob auch irgendwie die Ruhezeiten usw. eingehalten werden. Da kann man dann natürlich am nächsten Tag darauf hinweisen wenn „Du hast ja gar nicht auf die Ruhezeiten geachtet.“ oder „Du hast mehr als 10 Stunden am Stück gearbeitet, das geht doch so nicht!“ aber, viel mehr kann man auch nicht tun. | Nobody is instructed to work outside normal working hours, not at all. I don't expect that, and I don't expect that at home either. But if you want to do it because it's beneficial for one, then one should do it. But of course, one then has less influence on whether the rest periods etc. are adhered to. One can of course point this out the next day if "You didn't pay any attention to the rest periods" or "You worked more than 10 hours in a row, that's not possible!" but there's not much more you can do. |
| Role modeling | B6_SDG_13 | Tatsächlich habe ich mir wenig Gedanken dazu gemacht, wie es mir in dieser Situation geht. Ich bin da relativ straight aufgestellt. Das ist eine neue Situation, ich habe Verantwortung und man erwartet von mir, dass ich mich für die Mitarbeiter darauf einstelle. Ich habe mir da noch keine großen Gedanken zu gemacht. | **In fact, I haven't given much thought to how I feel in this situation.** I have a relatively straightforward approach. It's a new situation, I have responsibility and I'm expected to adapt to it for the employees. I haven't given it much thought yet. |
| Role modeling | B2_SDG_31 | Also da (Anm.: in Präsenz], da kann man, ist glaube ich so ne, so ne Vorbildfunktion eher darstellbar als jetzt über die Distanz des mobilen Arbeitens. Also das (ähm) wie gesundheitsorientierte Verhalten beim mobilen Arbeiten spielt aus meiner Perspektive, oder hat bei uns so keine Rolle gespielt. | So there [note: onsite], there one can, I think it's easier to act as a role model than now via the distance of mobile working. **So, from my perspective, the (um) health-oriented behavior in mobile working doesn't play or didn't play a role for us**. |
| Role modeling | A4_SK_50 | Da ist für mich immer die Frage: Was verschicke ich denn? Also, was ich für mich abarbeite ist ja eine Sache, aber wenn ich andere Personen mit einbinde, dann überlege ich mir durchaus wann ich das mache. Also ich schicke zum Beispiel nicht am Wochenende irgendwelche Mails, die ich auch Sonntagabend um 21 Uhr noch raus schicken kann, weil da liest sie eh keiner mehr zum Beispiel, oder Montagmorgen einfach. | The question for me is always: what do I send? Well, it's one thing what I do for myself, but when I involve other people, I think about when I do it. For example, **I don't send any emails at the weekend that I can still send out on Sunday evening at 9 p.m., because nobody reads them then anyway**, for example, or simply on Monday morning. |
| Role modelling | B10_SDG_24 | Dass ich da schon noch drauf achte, dass es mir gut geht und meine Ressourcen schone an der Stelle. Da hatte ich früher schon mal Probleme mit, deswegen achte ich da halt jetzt besonders drauf, dass es halt passt. | I still make sure that I'm doing well and that I'm conserving my resources in this area. I've had problems with that in the past, so now I pay particular attention to making sure it fits. |
| Role modeling | D9_EA_33 | Meine eigene Gesundheit ist, ich muss ein bisschen besser essen auch, aber jetzt von der Arbeitsbelastung habe ich da für mich selber keine Grenzen. Mit den Kollegen nehme ich es komplett anders. Da ist um 18:00 Uhr Schluss. Also, dass ich jemanden bitte, dass er mir um 18:00 Uhr oder nach 18:00 Uhr noch etwas hilft oder liefert, das ist seltenst. Ja das ist da bin ich hundertprozentig dahinter. Wenn Leute krank sind, dann sollen sie lieber länger daheimbleiben. Also da bin ich hundertprozentig dahinter, dass die Leute auskurieren und auch eine gute Work Life Balance haben. Mir bezüglich ist des komplett egal. Ich bin krasses Arbeitstier. | My own health is, I need to eat a bit better, too, but now with the workload I have no limits for myself. I take a completely different approach with my colleagues. They finish at 18:00. It's rare that I ask someone to help me or deliver something at 6 pm or after 6 pm. Yes, I'm one hundred percent behind that. If people are ill, they should stay at home longer. So, I'm one hundred percent behind people recovering and having a good work-life balance. I couldn't care less about that. I'm a massive workaholic. |

*Note.* Some quotes have been shortened for inclusion in the text (highlighted in bold).
